# Synthesis of different glutathione–sulfur mustard adducts of verified and potential biomarkers†

**DOI:** 10.1039/c8ra03360a

**Published:** 2018-06-29

**Authors:** Andreas Bielmann, Nicolas Sambiagio, Nathalie Wehr, Sandrine Gerber-Lemaire, Christian G. Bochet, Christophe Curty

**Affiliations:** Spiez Laboratory Austrasse, 3700 Spiez Switzerland christophe.curty@babs.admin.ch; University of Fribourg, Departement of Chemistry Chemin du Musée 9 1700 Fribourg Switzerland; École Polytechnique Fédérale de Lausanne, Institute of Chemical Sciences and Engineering Station 6 1015 Lausanne Switzerland

## Abstract

Sulfur Mustard (SM) is a blistering agent used as a chemical weapon. Glutathione (GSH) is involved in the β-lyase degradation pathway of SM and recently, bioadducts between SM and GSH were observed *in vitro*. While these bioadducts have never been isolated from *in vivo* tests or real poisoning with SM, they could be of interest as potential future biomarkers for the retrospective validation of exposure. We herein report the synthesis of different observed and new potential GSH–SM bioadducts as reference materials for analytical investigation. Two distinct approaches were investigated: a building-block pathway and the direct reaction with GSH. The availability of these references will aid future studies and may lead to the discovery of new GSH–SM biomarkers.

## Abstract 2

Recently, several adducts between the chemical agent sulfur mustard (SM) and glutathione (GSH) were observed *in vitro*. We report the synthesis of different observed and potential GSH–SM bioadducts as reference materials for analytical investigation.

## Graphical abstract

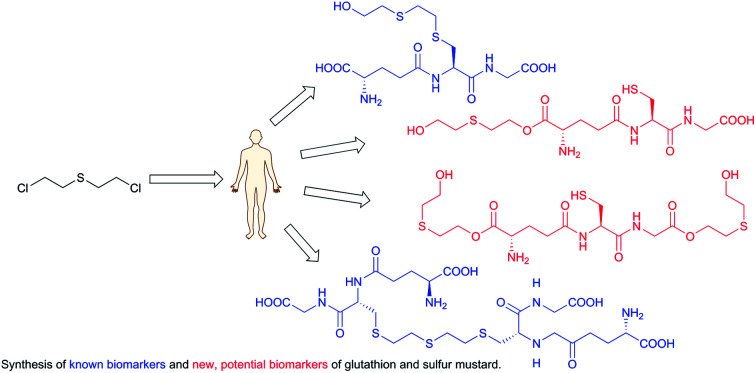

## Introduction

Sulfur Mustard (SM) or 2,2′-dichloroethyl sulfide was synthesized for the first time in 1822 by Despretz and its harmful properties were described 38 years later by Guthrie.^1^

SM was used as a chemical weapon for the first time in 1917 during the battle of Ypres, Belgium, which led to the name Yperite. During the 20^th^ century, SM was used on several occasions, most prominently during the Iraq–Iran War and in 2015 and 2016 during the Syrian Civil War.^2^ In 1997, the development, production, stockpile and use of SM and other chemical weapons were prohibited under the Chemical Weapons Convention (CWC), which is enforced by the Organisation for the Prohibition of Chemical Weapons (OPCW).^3^

SM is hydrophobic and can therefore easily pass through the skin and lipid cell membranes. Upon contact, SM acts as an irritant and after a latency period of 2 to 24 hours, blisters occur on the skin, which can turn into skin necrosis. Heavy injuries can occur with eye contact. The most dangerous form of contact however, is by inhalation. Respiratory tracts and lungs are damaged, which can lead to pulmonary edemas, the main cause of death after SM exposure. Treatment is purely symptomatic. Antibiotics are given to support the weakened immune system. The mortality rate after SM exposure is low, however, already 0.01 mg cm^−2^ of liquid contact on the skin leads to cutaneous redness and 0.5 mg cm^−2^ leads to the formation of huge vesicles.^4^

**Scheme 1:**
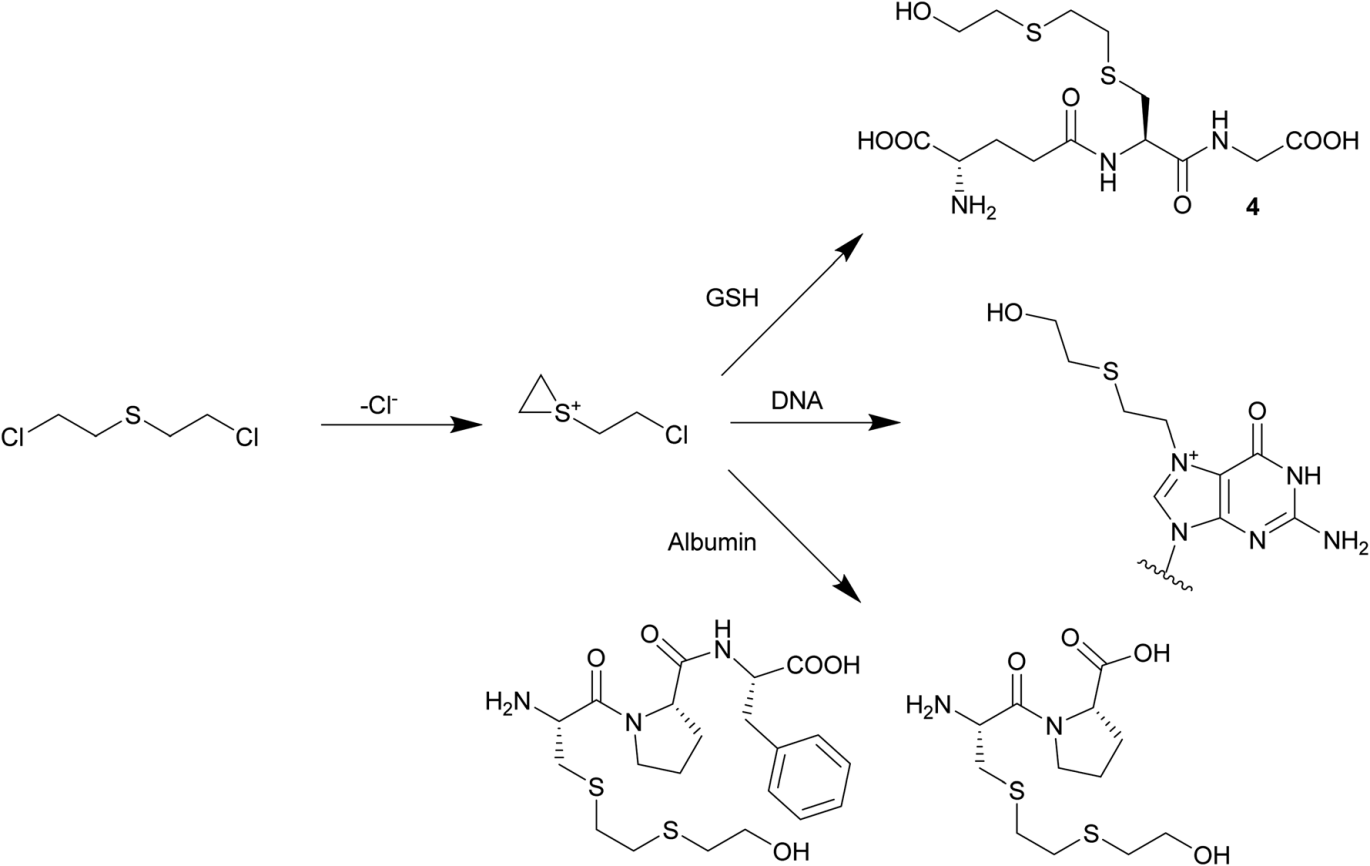
Formation of the reactive episulfonium ion and examples of bioadducts formed: *S*-HETE–glutathione, HETE–*N*7-guanine and C(HETE)P or C(HETE)PF, peptides of an albumin digest.^6^

The reactive alkylating species is generated by intramolecular cyclisation of SM and the formation of an episulfonium ion (Scheme 1). It alkylates cellular DNA and can cross-link DNA strands (intrastrand and interstrand), which inhibits DNA replication and leads to cell death. This is also believed to be the source of the latency period, which corresponds to the time the cell needs for a division. Alkylation of DNA through SM is believed to have long term adverse effects like cancers, chronic respiratory diseases and neurological disorders.^4*a*,5^

SM does not only form adducts with DNA, but also with other biomolecules like proteins or phospholipids. These adducts can be used as biomarkers to retrospectively give evidence for exposure.^7^ Bioadducts of DNA are the most studied. They either contain a 2-(2-(hydroxy)ethylthio)ethyl (HETE) moiety after alkylation and hydrolysis or are cross-linked with a 2-(ethylthio)ethyl (ETE) linker. Experiments with DNA incubated with ^35^S-labelled SM showed that DNA adducts are mainly formed with guanine (60%) and adenine (8%). Also, cross-linked guanine was observed (16%). These adducts have been synthesized to develop analytical methods and were analyzed by LC-MS/MS.^8^ Recently, an adduct between guanine, SM and glutathione (GSH) was discovered in mice exposed to SM, which could be observed by HPLC-MS/MS up to two weeks after exposure.^9^ Bioadducts are also formed with proteins like hemoglobin, albumin, globin and keratin, which can be detected after isolation and digestion with proteases. Adduct formation happens on the nucleophilic sites of the proteins. Alkylation of Val, His, Asp, Cys and Glu has been observed.^10^

GSH is a tripeptide with the formula γ-Glu-Cys-Gly. It plays a major role as a redox buffer in cells and is involved in detoxification and elimination of radicals, heavy metals and alkylating agents. GSH appears in high concentration in the liver (5 mM), but it is also transported into the blood stream (1 mM).^11^ GSH is involved in the degradation and excretion of SM by the glutathione/β-lyase pathway.^12^ Recently, several GSH–SM adducts were identified in *in vitro* tests. Black observed *S*-HETE–GSH in human blood incubated with SM.^10*a*^ Siegert observed the same adduct after direct treatment of GSH with SM.^13^ Halme incubated liver cytosol media in a phosphate buffer with SM and discovered the formation of *S*-HETE–GSH, GSH–ETE–GSH and 2-((2-(*S*-glutathionyl)ethyl)thio)ethyl phosphate. However, the last adduct might be formed due to reaction with the buffer phosphate.^14^ The above mentioned GSH–SM adducts were all detect by LC-MS.

The high concentration of GSH in the body and its affinity to react with SM makes the resulting adducts attractive candidates as biomarkers. However, none of these adducts have ever been isolated and characterized by analytical techniques other than LC-MS/MS. We herein present the synthesis and characterization of *S*-HETE–GSH and GSH–ETE–GSH which will help to determine their potential as biomarkers. Further, we present the synthesis of the potential bioadducts bis-*O*-HETE–GSH and *O*-HETE–GSH which so far have never been observed. Having these compounds available as references might support further analytical work to establish new biomarkers for the intoxication with SM.

## Results and discussion

### Synthesis of *S*-HETE–GSH 4

The initial strategy investigated to synthesize *S*-HETE–GSH 4 was to alkylate GSH with the protected half mustard *t*BuOETECl 2. The alkylating agent was prepared by protecting one of the hydroxyl groups of thiodiglycol (TDG) with a *t*Bu-protecting group following a modified procedure of Noort.^15^ Isobutylene was bubbled through a solution of TDG and H_2_SO_4_ in DCM to yield *t*BuOETEOH 1. While the yield remained modest, yield and purity increased from 9% to 15% and from >90% to >98% respectively.

The OH group of 1 was then replaced by Cl by slow addition of thionyl chloride, which yielded *t*BuOETECl 2 in high yield (99%) and purity.^15^

GSH was alkylated with 2 under slightly basic conditions.^15^ The resulting *S-t*BuOETE–GSH 3 was purified by reversed phase flash chromatography and obtained in good yield and purity.

**Scheme 2:**
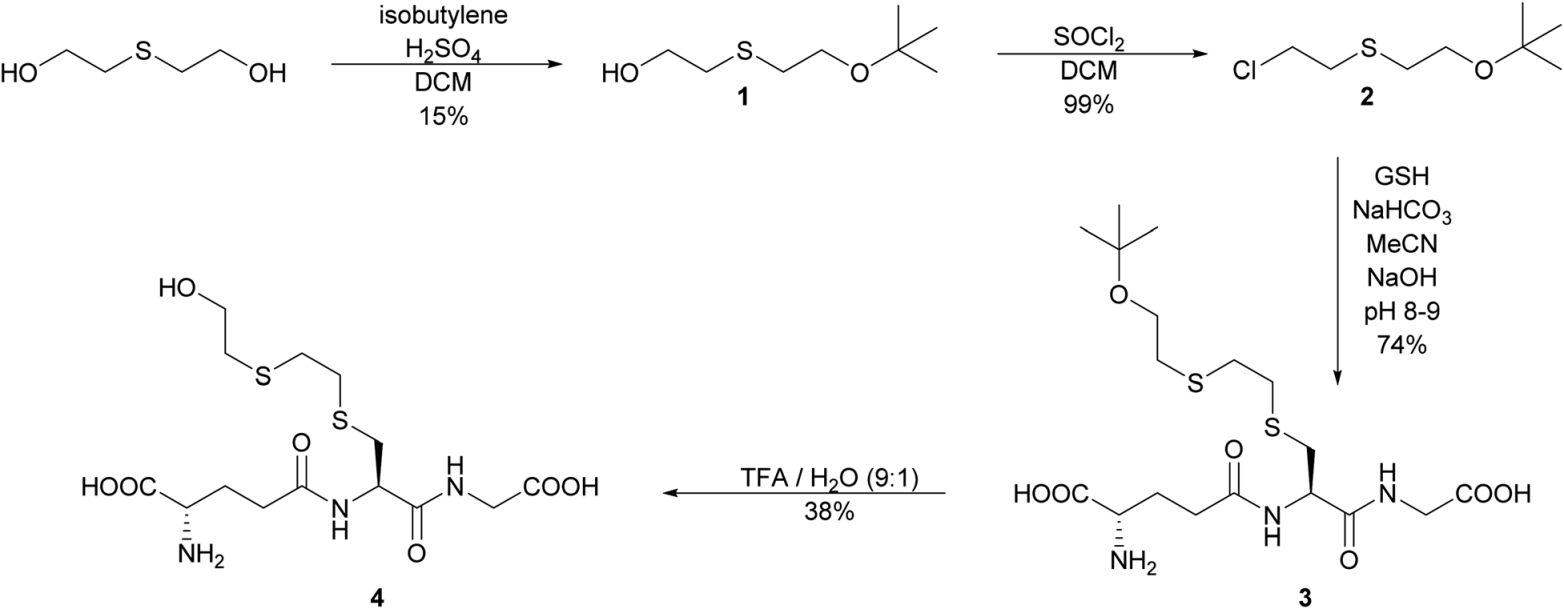
Synthesis pathway of *S*-HETE–GSH 4 by alkylation of glutathione.

Several conditions were tested to cleave the *tert*-butyl ether 3. Hydrolysis with phosphoric acid led to acceptable yield, however, the acid showed to be inseparable from the product.^16^ The use of Amberlyst 15 gave low yield and purity.^17^ The best result was obtained by using 90% TFA in aqueous solution. Some epimerization occurred, but the diastereomers could be separated, and *S*-HETE–GSH 4 was obtained in 38% yield and 95% purity (Scheme 2).

**Scheme 3:**
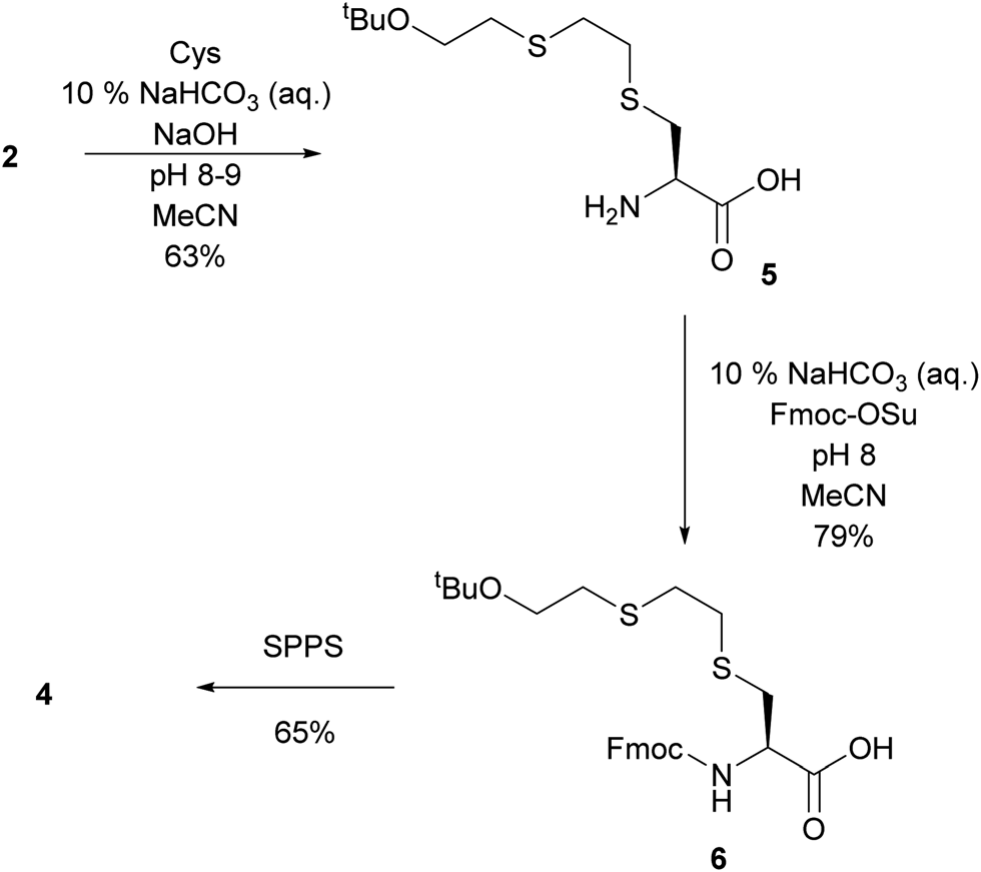
Synthesis of *S*-HETE–GSH 4 by SPPS.

As an alternative, a building-block approach was investigated in which cysteine was first alkylated and then used in solid-phase-peptide-synthesis (SPPS). l-Cysteine was alkylated with 2 using the same procedure which was previously used to alkylate GSH. *S-t*BuOETE–Cys 5 was obtained in 63% yield and very high purity. In the subsequent step the amine function of 5 was protected with Fmoc-OSu to give Fmoc-Cys(ETEO*t*Bu)–OH 6.^18^ SPPS was performed on preloaded Gly-2-CT polystyrene resin and DIC/Oxyma were used as coupling agents. In the Fmoc-deprotection steps 20% piperidine in DMF was used. Coupling of 6 proved to be slow and its reaction time needed to be increased to 3 h. Cleavage from the resin and deprotection were achieved with a cocktail consisting of TFA : TIS : H_2_O (95 : 2.5 : 2.5). After purification by precipitation in cold ether and subsequent flash chromatography 4 was obtained in 65% yield and >97% purity (Scheme 3).

With the direct alkylation approach 4 was obtained in two steps from GSH with 28% yield and >95% purity. The building-block approach achieved 36% yield (>97% purity) in three steps from l-cysteine. The building-block pathway is recommended since it provides the reference material 4 in better overall yield and purity.

### Synthesis of GSH–ETE–GSH 7

**Scheme 4:**
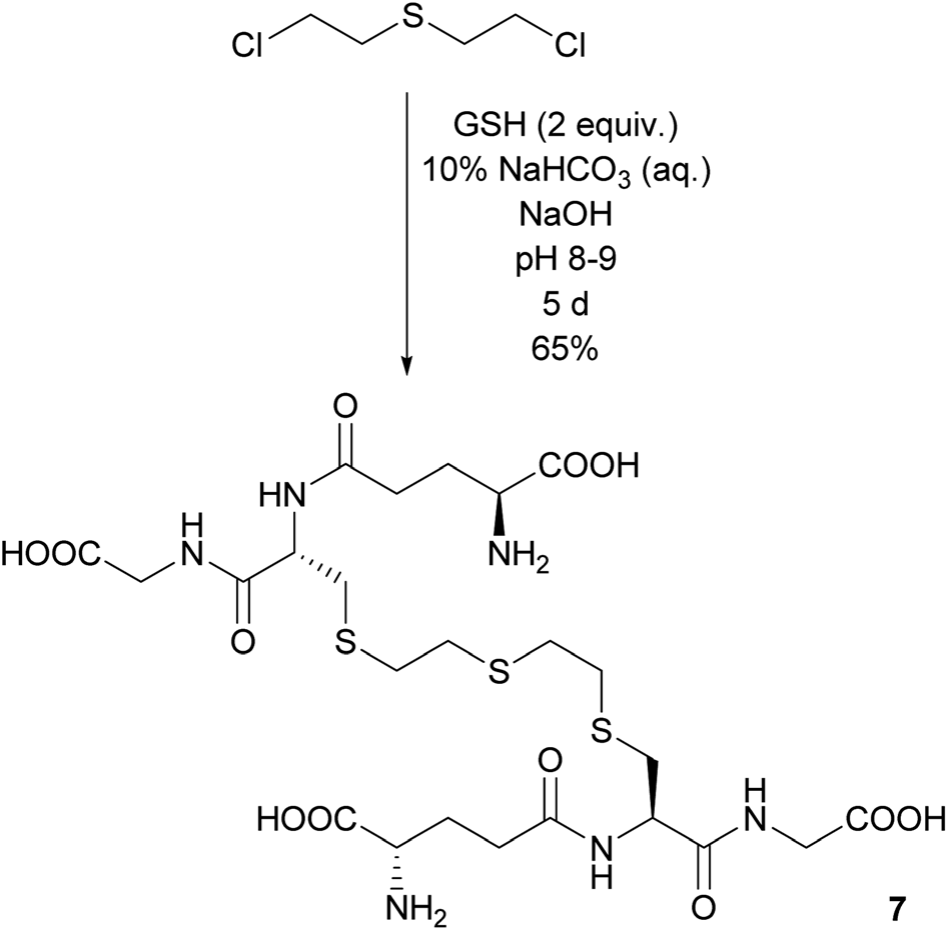
Dimerization of two glutathione molecules with sulfur mustard as linker.

Another compound, which has been observed in *in vitro* biological assays but has never been isolated, is the adduct consisting of two GSH linked by a sulfur mustard moiety *via* their thiol functions. Indeed, when a twofold excess of GSH reacted under slightly basic conditions with one equivalent of sulfur mustard, GSH–ETE–GSH 7 was formed. The reaction proceeded slowly, but after five days 7 could be isolated in 65% yield in 85% purity. The impurity was identified as residual acetonitrile (Scheme 4).

### Synthesis of bis-*O*-HETE–GSH 8

**Scheme 5:**
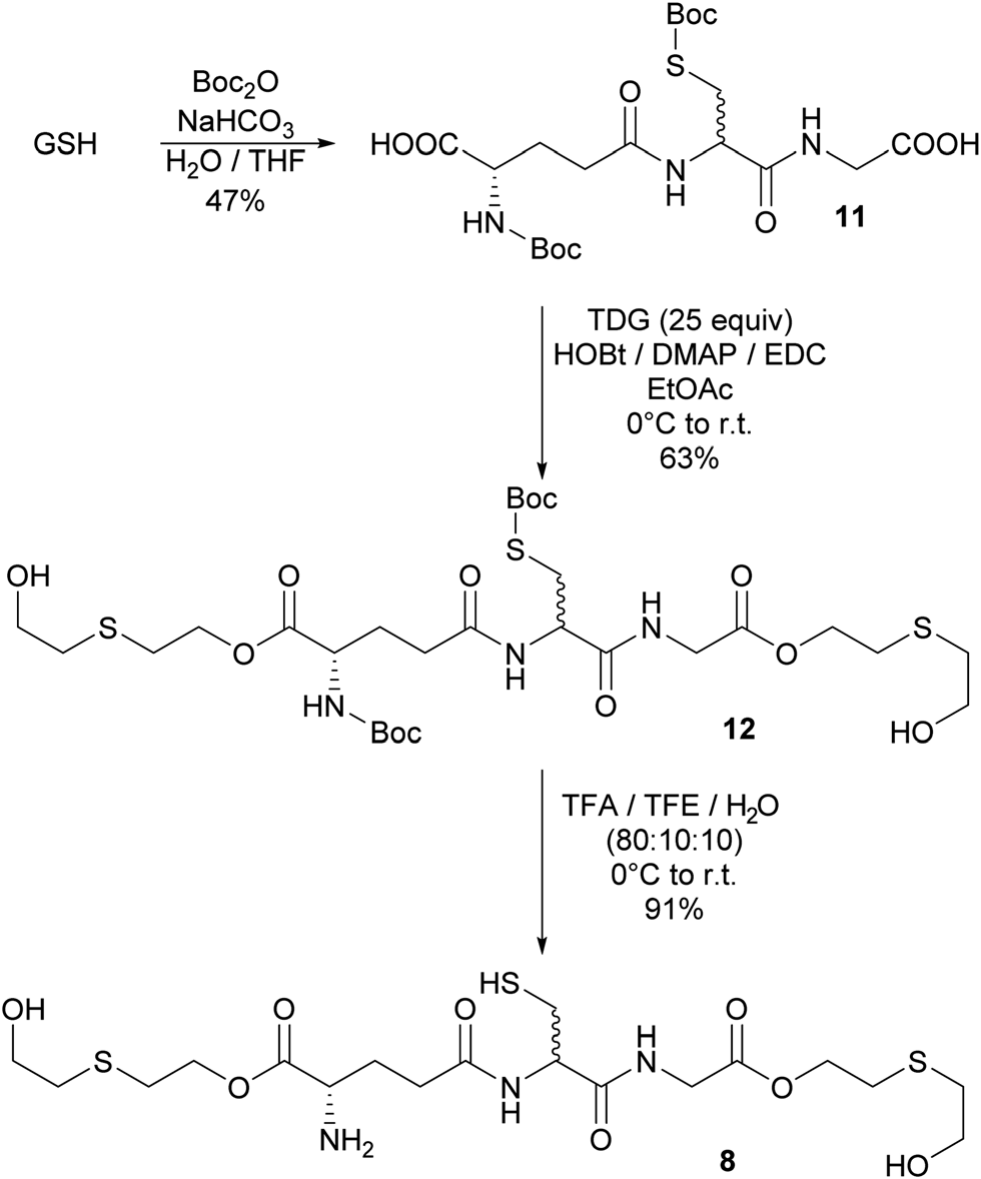
Synthesis of bis-*O*-HETE–GSH 8.

The bioadducts bis-*O*-HETE–GSH 8, *O*-HETE–GSH 9 and GSH–*O*-HETE 10 have never been observed. However, SM adducts of glutamic acid and aspartic acid have been observed *in vitro*, after SM reacted with their side chain acid groups.^10*a*^ Therefore it could be envisageable that the adducts 8, 9 and 10 would be formed as potential biomarkers. Both amine and thiol functionalities of GSH were protected in the presence of Boc_2_O to increase its solubility in organic solvents.^19^ Protected 11 still contained *S*-Boc and *N*-Boc monoprotected GSH as minor byproducts. 11 was observed as two diastereomers in a 1 : 1 ratio. Epimerization occurred at the cysteine moiety due to the basic conditions of the coupling. Several conditions to esterify 11 with excess of TDG were screened (Table 1). Best results for the coupling were obtained using EDC, HOBt and DMAP under inert atmosphere (Table 1, entry 10). However, the bis-*O*-HETE-*N*,*S*-Boc-GSH adduct 12 could only be obtained in moderate purity. It was engaged without further purification in the subsequent deprotection step using a solution of TFA, TFE and water. While high yield was achieved, the final product 8 could not be separated from a side product which was identified as the *tert*-butyl thioether of 8. Neither could the diastereoisomers be separated. This resulted in reduced purity of >75% (Scheme 5).

**Table 1:** Coupling conditions for the synthesis of bis-*O*-HETE-*N*,*S*-Boc-GSH 12

Entry	Coupling Agents	Bases	*T*/°C	*t*/h	Equiv. TDG	Yield/%	Purity/% (H NMR)
1	EDC	—	0 °C – >r.t.	24	13	19	95
2	EDC	—	0 °C – >r.t.	22	26	13	50
3	EDC/HOBt	—	0 °C – >r.t.	22	26	50	70
4	EDC/HOBt	DMAP	0 °C – >r.t.	22	26	55	70
5	EDC/HOBt	TEA	0 °C – >r.t.	22	26	40	57
6	EDC/HOBt	DMAP/TEA	0 °C – >r.t.	22	26	40	59
7	EDC	DMAP	0 °C – >r.t.	22	26	34	54
8	EDC	DMAP/TEA	0 °C – >r.t.	22	26	24	63
9	EDC	TEA	0 °C – >r.t.	22	26	7	38
10	EDC/HOBt^a^	DMAP	0 °C – >r.t.	22	26	69	68

a Reaction conducted under inert atmosphere.

### Synthesis *O*-HETE–GSH

**Fig. 1:**
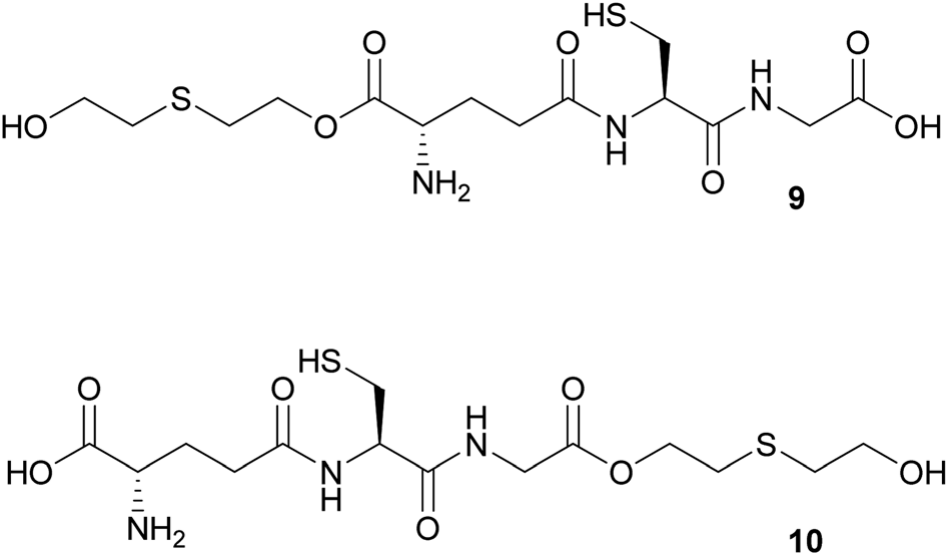
The two possibilities for *O*-alkylated GSH: *O*-HETE–GSH 9 and GSH–*O*-ETEH 10.

In the attempt to synthesize the mono-*O*-HETE–GSH adducts 9 and 10 (Fig. 1), 11 was subjected to the coupling conditions of Table 1, entry 4, but only one equivalent of TDG was used. LC-HRMS analysis of the resulting product mixture revealed that *ca.* 70% corresponded to either one of the two possible mono-*O*-HETE-*N*,*S*-Boc-GSH compounds, while *ca.* 11% corresponded to the bis-*O*-HETE-*N*,*S*-Boc-GSH 12 (data not shown). The mixture could not be separated. The experiment showed that, as expected, there was no selectivity between the two C-termini of 11.

**Scheme 6:**
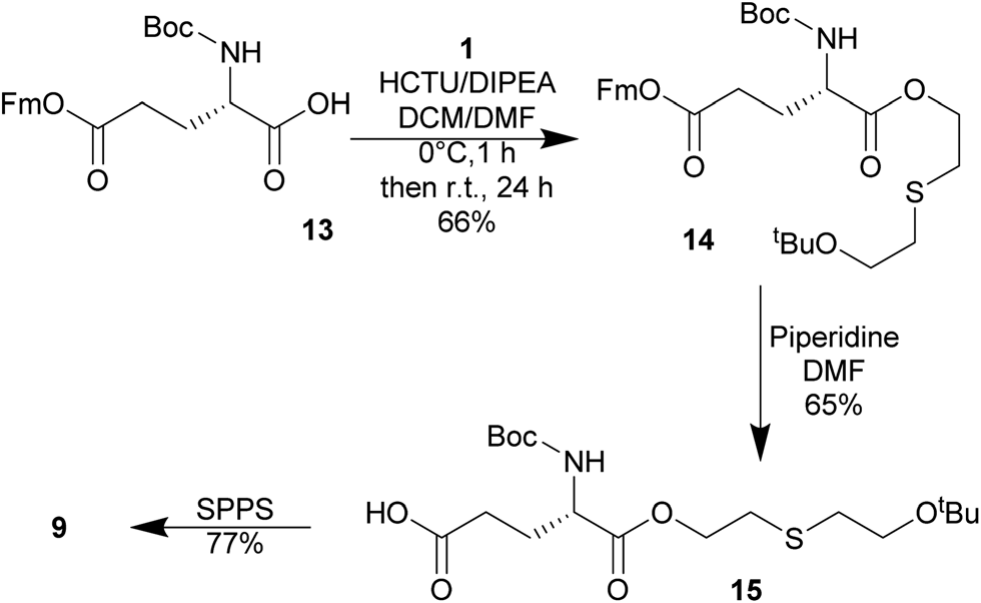
Synthesis of *O*-HETE–GSH 9 using a building-block strategy.

Therefore, a building-block approach to synthesize 9 was investigated instead. Commercially available Boc-Glu(OFm)–OH 13 was esterified with 1 using HCTU/DIPEA which gave Boc-Glu(OFm)-OETEO*t*Bu 14. The Fm group was selectively cleaved with 20% piperidine in DMF giving Boc-Glu(OH)-*O*-ETEO*t*Bu 15 in 68% yield and good purity. 15 was used in SPPS to give 9 in 77% yield and >90% purity (Scheme 6). The synthesis of 10 was not attempted.

## Conclusion

Several synthesis strategies were explored to produce sulfur mustard–glutathione adducts as potential biomarkers for retrospective validation of exposure to this blistering agent. *S*-HETE–GSH 4 was successfully synthesized. Two pathways towards 4 were investigated. The direct alkylation of GSH with 2 and subsequent deprotection led to 28% overall yield from GSH and >95% purity. However, better yield and purity were obtained when using a building-block approach and SPPS to construct the final peptide. While requiring an additional step, this route yielded 36% overall yield from Fmoc-Cys-OH and >97% purity. It is recommended to use the building-block pathway to obtain 4, since the reference material can be obtained in better overall yield and purity.

The dimer GSH–ETE–GSH 7 was obtained in one step by condensing GSH with sulfur mustard.

The potential biomarker bis-*O*-HETE–GSH 8 was synthesized directly from the reaction of SM with GSH. While 8 was only obtained in moderate purity, the characterization data will help to investigate its presence in future *in vitro* and *in vivo* screenings.

Using the protected precursor Boc-Glu(OH)-*O*-ETEO*t*Bu 15 in SPPS allowed the synthesis of the mono-*O*-HETE–GSH derivative 9 with 77% yield and >90% purity in three steps.

## Experimental part

Unless otherwise stated, all reagents were purchased from Sigma-Aldrich and were used without further purification. Glutathione was obtained from Iris Biotech. Deuterated solvents were purchased from Armar AG and Cambridge Isotope Laboratories, Inc. Boc-Glu(OFm)–OH was purchased from Bachem. Sulfur mustard was synthesized and provided by Spiez Laboratory. **CAUTION**: sulfur mustard is a schedule 1 chemical and is highly toxic. Adequate protection is needed and appropriate safety measures have to be taken when handling this compound.

Thin layer chromatography (TLC) was performed on silica gel 60 F-254 pre-coated aluminum sheets thin layer chromatography plates and silica gel RP-18 F-254S pre-coated aluminum sheets TLC plates from Merck.

Reaction monitoring by mass analysis was done by direct injection into a Dalton Mass Detector (ESI) from Biotage.

Flash column chromatography was carried out with an Isolera One system coupled with a Dalton Mass Detector from Biotage. Biotage SNAP Ultra cartridges (10 g, 25 g, 50 g and 100 g) and Biotage SNAP Ultra C18 cartridges (12 g, 30 g and 60 g) were used.

SPPS was performed on an Initiator+ Alstra automated peptide-synthesizer from Biotage.

LC-HRMS (ESI) analyses were done on an Agilent Technologies 1290 Infinity LC System instrument with a Bruker Daltonics maXis UHR QTof 4G MS. As a column the Sigma-Aldrich Discovery HS C18 (150 mm × 2.1 mm, particle size 5 μm) was used. As eluents H_2_O with 5 mM NH_4_Ac and MeOH with 5 mM NH_4_Ac were used with a flow-rate of 0.6 mL min^−1^.

GC-MS (EI) analyses were performed on an Agilent Technologies 7890A instrument coupled with an Agilent Technologies 5975C inert MSD. The measurements were performed with the HP-1701 (30 m × 0.25 mm × 0.25 μm, 14% cyanopropyl-phenyl/86% PDMS) high resolution gas chromatography column using a temperature program (40 °C for 3 min, 13 °C min^−1^ until 280 °C and 280 °C for 3.54 min). The injector and the detector temperatures were 220 °C and 250 °C, respectively. The splitless injection mode was used to inject volumes of 1 μL (*c* = 0.5 mg mL^−1^). Helium was used as carrier gas (1 mL min^−1^).

NMR spectra were recorded on a Bruker Avance III HD 400 MHz Nano Bay spectrometer in CDCl_3_, DMSO-d_6_, MeOD or D_2_O. ^1^H NMR spectra were measured at 400 MHz and ^13^C NMR spectra at 100 MHz. Chemical shifts are expressed as parts per million (*δ*) using TMS or residual solvent protons as internal standards. Coupling constants (*J*) are reported in Hz. Splitting patterns are described as s (singlet), br. (broad singlet), d (doublet), dd (double doublet), dt (double triplet), t (triplet), td (triple doublet), q (quartet) and m (multiplet).

IR spectra were measured on a Bruker Tensor 27 and a Jasco FT/IR-4100 spectrometer.

Purities were assessed by NMR.

### 2-(2-(*tert*-Butoxy)ethylthio)ethanol, *t*BuOETEOH 1

Thiodiglycol (4.60 mL, 45 mmol, 1 equiv.) was dissolved in DCM (45 mL) and H_2_SO_4_ (0.27 mL, 5 mmol) was added. The reaction mixture was cooled to 4 °C and isobutylene was passed through the solution for 1.5 h. The solution was stirred for 72 h at room temperature. The reaction was monitored by GC-MS. The mixture was washed with H_2_O (3 × 25 mL) and sat. aq. NaHCO_3_ (1 × 15 mL). The organic layer was dried over MgSO_4_ and the solvent was evaporated under reduced pressure affording a colorless oil (1.18 g, 7 mmol, 15%, purity > 98%).

^1^H NMR (400 MHz, CDCl_3_) *δ*: 3.77 (dt, *J* = 6 Hz, 2H, C*H*_2_OH), 3.55 (t, *J* = 7 Hz, 2H, C*H*_2_O*t*Bu), 3.16 (t, *J* = 7 Hz, 1H, O*H*), 2.78 (t, *J* = 6 Hz, 2H, C*H*_2_CH_2_OH), 2.73 (t, *J* = 7 Hz, 2H, C*H*_2_CH_2_O*t*Bu), 1.22 (s, 9H, C(C*H*_3_)_3_) ppm.

^13^C NMR {^1^H} (100 MHz, CDCl_3_) *δ*: 73.6 (*C*(CH_3_)_3_), 62.1 (*C*H_2_O*t*Bu), 61.4 (*C*H_2_OH), 36.3 (*C*H_2_CH_2_OH), 32.6 (*C*H_2_CH_2_O*t*Bu), 27.5 (C(*C*H_3_)_3_) ppm.

GCMS (EI) RT: 13.0 min, *m*/*z*: [M]^+^ 178 (0.2%), [M^+^ − H_2_O] 160 (9%), [M^+^ − OC(CH_3_)_3_] 105 (33%), [M^+^ − CHOC(CH_3_)_3_] 92 (30%), [HSCH_2_CH_2_]^+^ 61 (17%), [C(CH_3_)_3_]^+^ 57 (100%).

FTIR (neat): 3456, 3419, 3401, 3374, 2973, 2929, 2872, 2254, 2242, 2217, 2198, 2176, 1959, 1947, 1654, 1592, 1473, 1391, 1363, 1284, 1260, 1233, 1194, 1069, 1045, 1016, 883, 826, 777, 692 cm^−1^.

### 2-(2-(*tert*-Butoxy)ethylthio)ethyl chloride, *t*BuOETECl 2

1 (1.45 g, 8 mmol) was dissolved in DCM (15 mL) and a solution of SOCl_2_ (830 μL, 11 mmol, 1.4 equiv.) in DCM (10 mL) was added dropwise at 0 °C. The reaction was monitored by GC-MS, and after complete conversion of the starting material, the solvent was evaporated under reduced pressure. The product was obtained as a yellowish oil (1.58 mg, 8 mmol, 99%, purity > 98%).

^1^H NMR (400 MHz, CDCl_3_) *δ*: 3.67 (t, *J* = 7.8 Hz, 2H, C*H*_2_Cl), 3.55 (t, *J* = 6.5 Hz, 2H, C*H*_2_O*t*Bu), 2.94 (t, *J* = 8.0 Hz, 2H, C*H*_2_CH_2_Cl), 2.70 (t, 2H, *J* = 6.5 Hz, C*H*_2_CH_2_O*t*Bu), 1.20 (9H, s, C(*C*H_3_)_3_) ppm.

^13^C NMR {^1^H} (100 MHz, CDCl_3_) *δ*: 73.3 (*C*(CH_3_)_3_), 62.2 (*C*H_2_O*t*Bu), 43.2 (*C*H_2_Cl), 34.8 (*C*H_2_CH_2_Cl), 33.0 (*C*H_2_CH_2_O*t*Bu), 27.5 (C(*C*H_3_)_3_) ppm.

GC-MS (EI) RT: 12.8 min, *m*/*z*: [M^+^ − HCl] 160 (15%), [M^+^ − OC(CH_3_)_3_] 123 (31%), [M^+^ – CHOC(CH_3_)_3_] 110 (32%), [HSHCH_2_CH_2_]^+^ 61 (14%), [C(CH_3_)_3_]^+^ 57 (100%).

FTIR (neat): 3789, 3658, 2904, 1622, 1409, 1252, 1072, 886, 678 cm^−1^.

### *S*-(2-(2-(*tert*-Butoxy)ethylthio)ethyl)glutathione, *S-t*BuOETE–GSH 3

To a solution of GSH (470 mg, 1.53 mmol, 1 equiv.) in sat. aq. NaHCO_3_ (10 mL) was added portionwise a solution of 2 (400 mg, 2.03 mmol, 1.3 equiv.) in MeCN (10 mL). The pH of the solution was kept at 8–9 by the addition of aq. NaOH (0.1 M). The mixture was stirred for 2 h and monitored by MS. The mixture was washed with DCM (3 × 12 mL) and the aqueous layer was concentrated under reduced pressure. The crude product was purified by reversed phase flash chromatography (SNAP Ultra C18 60 g, H_2_O/MeCN from 0% to 100% MeCN). The product was obtained as a colorless solid (532 mg, 1.14 mmol, 74%, purity > 98%).

^1^H NMR (400 MHz, D_2_O) *δ*: 4.61–4.58 (m, 1H, Cys C*H*CH_2_), 3.78 (d, *J* = 8 Hz, 2H, Gly C*H*_2_COOH), 3.65 (t, *J* = 6 Hz, 2H, C*H*_2_O*t*Bu), 3.46 (t, *J* = 7 Hz, 1H, Glu C*H*NH_2_), 3.11 (dd, *J* = 5 Hz, *J* = 14 Hz, 1H, Cys CHC*H*_2_S), 2.89 (dd, *J* = 9 Hz, *J* = 14 Hz, 1H, Cys CHC*H*_2_S), 2.85 (m, 4H, SC*H*_2_C*H*_2_S), 2.74 (t, *J* = 6 Hz, 2H, C*H*_2_CH_2_O*t*Bu), 2.50–2.40 (m, 2H, Glu CH_2_C*H*_2_CONH), 2.08–1.86 (m, 2H, Glu C*H*_2_CH_2_CONH), 1.24 (s, 9H, C(C*H*_3_)_3_) ppm.

^13^C NMR {^1^H} (100 MHz, D_2_O) *δ*: 179.2 (*C*O), 176.2 (*C*O), 175.8 (*C*O), 171.9 (*C*O), 75.2 (*C*(CH_3_)_3_), 61.0 (*C*H_2_O*t*Bu), 55.0 (Cys *C*HCH_2_), 53.1 (Glu *C*HNH_2_), 43.4 (Gly *C*H_2_COOH), 32.9 (*C*H_2_CH_2_O*t*Bu), 32.0 (Cys CH*C*H_2_S), 31.6 & 31.5 (S*C*H_2_*C*H_2_S), 31.3 (Glu CH_2_*C*H_2_CONH), 29.1 (Glu *C*H_2_CH_2_CONH), 26.6 (C(*C*H_3_)_3_) ppm.

HRMS (ESI/Q-TOF) *m*/*z* [M + H]^+^ calcd for C_18_H_34_N_3_O_7_S_2_ 468.1832, found: 468.1822.

FTIR (neat): 2975, 1642, 1586, 1390, 1361, 1307, 1259, 1233, 1196, 1092, 1072, 1018, 911, 883, 824, 641 cm^−1^.

Mp 196 °C (degradation).

### *S*-(2-(2-(Hydroxy)ethylthio)ethyl)glutathione, *S*-HETE–GSH 4 by alkylation of GSH

A solution of 90% aq. TFA was slowly added to 3 (347 mg, 0.74 mmol, 1 equiv.) at 0 °C. The reaction mixture was then stirred at room temperature for 2 h. TFA and water were evaporated under reduced pressure and the crude product was purified by flash chromatography (SNAP Ultra 25 g, DCM/MeOH from 21% to 100% MeOH). The product was obtained as a viscous colorless oil (116 mg, 0.28 mmol, 38%, purity > 95%).

^1^H NMR (400 MHz, D_2_O) *δ*: 4.59 (dd, *J* = 4 Hz, *J* = 8 Hz, 1H, Cys C*H*CH_2_), 4.06 (t, *J* = 6 Hz, 1H, Glu C*H*NH_2_), 4.02 (s, 2H, Gly C*H*_2_COOH), 3.75 (t, *J* = 6 Hz, 2H, C*H*_2_OH), 3.09 (dd, *J* = 6 Hz, *J* = 14 Hz, 1H, Cys CHC*H*_2_S), 2.90 (dd, *J* = 9 Hz, *J* = 14 Hz, 1H, Cys C*H*CH_2_S), 2.84 (s, 4H, SC*H*_2_C*H*_2_S), 2.75 (t, *J* = 6 Hz, 2H, C*H*_2_CH_2_OH), 2.66–2.53 (m, 2H, Glu CH_2_C*H*_2_CONH), 2.31–2.16 (m, 2H, Glu C*H*_2_CH_2_CONH) ppm.

^13^C NMR {^1^H} (100 MHz, D_2_O) *δ*: 174.5 (*C*O), 172.9 (*C*O), 172.7 (*C*O), 172.2 (*C*O), 60.3 (*C*H_2_OH), 53.1 (Cys *C*HCH_2_), 52.3 (Glu *C*HNH_2_), 41.1 (Gly *C*H_2_COOH), 33.3 (*C*H_2_CH_2_OH), 32.7 (Cys CH*C*H_2_S), 31.6 & 31.0 (S*C*H_2_*C*H_2_S), 30.9 (Glu CH_2_*C*H_2_CONH), 25.5 (Glu *C*H_2_CH_2_CONH) ppm.

HRMS (ESI/Q-TOF) *m*/*z* [M + H]^+^ calcd for C_14_H_26_N_3_O_7_S_2_ 412.1207, found: 412.1202.

FTIR (neat): 1671, 1640, 1616, 1602, 1589, 1556, 1526, 1511, 1412, 1400, 1353, 1308, 1233, 1202, 1132, 1066, 1047, 1041, 1010, 916, 909, 877, 657 cm^−1^.

### *S*-(2-((2-(*tert*-Butoxy)ethyl)thio)ethyl)cysteine, *S-t*BuOETE-Cys 5

l-Cysteine (512 mg, 4 mmol, 1 equiv.) was dissolved in sat. aq. NaHCO_3_ (25 mL). A solution of 2 (1150 mg, 6 mmol, 1.5 equiv.) in MeCN (25 mL) was added portionwise over 30 min. The pH of the reaction mixture was kept at 8–9 by addition of aq. NaOH (0.1 M). The solution was stirred for 2 h at room temperature and monitored by TLC (DCM/MeOH, 7 : 3). The mixture was concentrated under reduced pressure and the crude product was purified by reversed phase flash chromatography (SNAP Ultra C18 60 g, H_2_O/MeCN from 0% to 60% MeCN). The product was obtained as a white powder (755 mg, 3 mmol, 63%, purity > 98%).

^1^H NMR (400 MHz, D_2_O) *δ*: 3.85 (dd, *J* = 4.3 Hz, *J* = 7.3 Hz, 1H, C*H*), 3.57 (t, *J* = 6.4 Hz, 2H, C*H*_2_O*t*Bu), 3.10–2.96 (m, 2H, CHC*H*_2_), 2.78 (s, 4H, SC*H*_2_C*H*_2_S), 2.66 (t, *J* = 6.4 Hz, 2H, C*H*_2_CH_2_O*t*Bu), 1.15 (s, 9H, C(C*H*_3_)_3_) ppm.

^13^C NMR {^1^H} (100 MHz, D_2_O) *δ*: 172.8 (*C*O), 75.2 (*C*(CH_3_)_3_), 60.9 (*C*H_2_O), 53.6 (*C*H), 32.0 (S*C*H_2_CH_2_S), 31.5 (SCH_2_*C*H_2_S), 31.3 (CH*C*H_2_), 31.1 (*C*H_2_CH_2_O*t*Bu), 26.5 (C(*C*H_3_)_3_) ppm.

HRMS (ESI/Q-TOF) *m*/*z* [M + H]^+^ calcd for C_11_H_24_NO_3_S_2_ 282.1192, found: 282.1188.

FTIR (neat): 3700, 2973, 2905, 1588, 1409, 1255, 1071, 886 cm^−1^.

### *N*-(((9*H*-Fluoren-9-yl)methoxy)carbonyl)-*S*-(2-((2-(*tert*-butoxy)ethyl)thio)ethyl)cysteine, Fmoc-Cys(ETEO*t*Bu)–OH 6

5 (1200 mg, 4 mmol, 1 equiv.) was dissolved in H_2_O (10 mL) and a solution of aq. Na_2_CO_3_ (10%) was added until pH 8. The solution was then cooled to 0 °C and a solution of Fmoc-OSu (2200 mg, 6 mmol, 1.5 equiv.) in MeCN (30 mL) was added dropwise over 30 min. The reaction mixture was stirred 1 h at 0 °C and at room temperature over night. The solvent was then removed under reduced pressure and H_2_O (15 mL) was added. The solution was acidified to pH 2 with a solution of KHSO_4_ (5%) and extracted with EtOAc (3 × 45 mL). The organic phases were combined and washed with brine (20 mL) and H_2_O (20 mL), dried over Na_2_SO_4_ and concentrated under reduced pressure. The crude product was purified by flash chromatography (SNAP Ultra 50 g, hexane/EtOAc from 10% to 60% EtOAc). The product was obtained as a white solid (1706 mg, 4 mmol, 79%, purity > 95%).

^1^H NMR (400 MHz, CD_3_CN) *δ*: 7.87 (d, *J* = 7.5 Hz, 2H, Fmoc), 7.71 (d, *J* = 7.4 Hz, 2H, Fmoc), 7.45 (t, *J* = 7.4 Hz, 2H, Fmoc), 7.37 (t, *J* = 7.4 Hz, 2H, Fmoc), 6.16 (d, *J* = 7.9 Hz, 1H, N*H*), 4.44–4.35 (m, 3H, Fmoc), 4.28 (t, *J* = 6.8 Hz, 1H, Fmoc C*H*), 3.51 (t, *J* = 6.5 Hz, 2H, C*H*_2_O*t*Bu), 3.12–2.87 (m, 2H, C(O)CHC*H*_2_), 2.78 (s, 4H, SC*H*_2_C*H*_2_S), 2.63 (t, *J* = 6.5 Hz, 2H, C*H*_2_CH_2_O*t*Bu), 1.16 (s, 9H, C(C*H*_3_)_3_) ppm.

^13^C NMR {^1^H} (100 MHz, CD_3_CN) *δ*: 144.7 (Fmoc), 141.7 (Fmoc), 128.3 (Fmoc), 127.8 (Fmoc), 125.8 (Fmoc), 120.6 (Fmoc), 73.5 (*C*(CH_3_)_3_), 62.3 (*C*H_2_O), 54.5 (C(O)*C*H), 47.6 (Fmoc CH), 32.9 (S*C*H_2_CH_2_S), 32.7 (SCH_2_*C*H_2_S), 32.6 (CH*C*H_2_), 27.3C(*C*H_3_)_3_ ppm.

HRMS (ESI/Q-TOF) *m*/*z* [M + H]^+^ calcd for C_26_H_34_NO_5_S_2_ 504.1872, found: 504.1879.

FTIR (neat): 3701, 2972, 2362, 1723, 1636, 1530, 1447, 1205, 1075, 74 cm^−1^.

### *S*-(2-(2-(Hydroxy)ethylthio)ethyl)glutathione, *S*-HETE–GSH 4 by SPPS

The synthesis of *S*-(HETE)-GSH 4 was performed by SPPS on a 0.2 mmol scale. The preloaded resin H-Gly-2-ClTrt resin (0.182 g, loading 1.1 mmol g^−1^) was swelled with DMF (4.5 mL) over 2 h at room temperature. The resin was then mixed with 6 (428 mg, 0.8 mmol, 4 equiv.) in DMF (1.35 mL), DIC (128 μL, 0.8 mmol, 4 equiv.) in DMF (1.52 mL) and Oxyma pure (117 mg, 0.8 mmol, 4 equiv.) in DMF (1.65 mL). The reaction mixture was stirred for 3 h at room temperature. After DMF washes, a solution of 20% piperidine in DMF (4.5 mL, 90 equiv.) was added twice, for 5 min and 15 min agitation respectively. After another washing step, Boc-Glu-O*t*Bu (234 mg, 0.8 mmol, 4 equiv.) in DMF (1.5 mL), DIC (128 μL, 0.8 mmol, 4 equiv.) in DMF (1.52 mL) and Oxyma pure (117 mg, 0.8 mmol, 4 equiv.) in DMF (1.65 mL) were added to the resin. The reaction mixture was stirred again for 3 h at room temperature, followed by a pre-cleavage wash of the resin with DCM (4 × 9 mL). An automated pre-cleavage wash with DCM (4.5 mL), MeOH (4.5 mL) and DCM (4.5 mL) was then performed by the instrument. The resin was transferred into a new flask and the following cleavage cocktail was added: TFA : TIS : H_2_O (10 mL, 95 : 2.5 : 2.5). The reaction mixture was stirred for 2 h at room temperature and the resin was then filtered and washed with TFA. The solution was concentrated under reduced pressure and the crude product was precipitated in ice cold Et_2_O. The final purification was performed by reversed phase flash chromatography (SNAP Ultra C18 12 g, H_2_O/MeCN from 0% to 60% MeCN). The product was obtained as white crystal (53.5 mg, 0.13 mmol, 65%, purity > 97%).

^1^H NMR (400 MHz, D_2_O) *δ*: 4.52 (q, *J* = 3 Hz, 1H, Cys C*H*CH_2_), 3.92 (s, 2H, Gly C*H*_2_COOH), 3.81 (t, *J* = 7 Hz, 1H, Glu C*H*NH_2_), 3.67 (t, *J* = 6 Hz, 2H, C*H*_2_OH), 3.04–2.83 (m, 2H, Cys CHC*H*_2_S), 2.81 (s, 4H, SC*H*_2_C*H*_2_S), 2.76 (t, *J* = 6 Hz, 2H, C*H*_2_CH_2_OH), 2.50 (q, *J* = 5 Hz, 2H, Glu CH_2_C*H*_2_CONH), 2.11 (t, *J* = 7 Hz, 2H, Glu C*H*_2_CH_2_CONH) ppm.

^13^C NMR {^1^H} (100 MHz, D_2_O) *δ*: 174.7 (*C*O), 173.14 (*C*O), 173.09 (*C*O), 172.7 (*C*O), 60.3 (*C*H_2_OH), 53.4 (Cys *C*HCH_2_), 53.1 (Glu *C*HNH_2_), 41.3 (Gly *C*H_2_COOH), 33.3 (*C*H_2_CH_2_OH), 32.7 (Cys CH*C*H_2_S), 31.6 & 31.1 (S*C*H_2_*C*H_2_S), 25.8 (Glu *C*H_2_CH_2_CONH) ppm.

HRMS (ESI/Q-TOF) *m*/*z* [M + H]^+^ calcd for C_14_H_26_N_3_O_7_S_2_ 412.1206, found: 412.1207.

FTIR (neat): 3335, 2532, 1644, 1516, 1414, 1229, 1022, 678 cm^−1^.

### Glutathione–ethylthioethyl–glutathione, GSH–ETE–GSH 7

To a solution of GSH (1.310 g, 4.26 mmol, 1 equiv.) in sat. aq. NaHCO_3_ (10 mL) was added dropwise a solution of sulfur mustard (0.323 g, 2.03 mmol, 0.5 equiv.) in MeCN (10 mL). The pH of the solution was kept at 8–9 by the addition of aq. NaOH (0.1 M). The mixture was stirred at room temperature until complete consumption of sulfur mustard (118 h, monitored by GC-MS) was observed. The mixture was washed with DCM (3 × 15 mL) and the aqueous layer was concentrated under reduced pressure. The crude product was purified by reversed phase flash chromatography (Biotage, SNAP Ultra C18 60 g, 25% to 95% H_2_O in MeCN). The solvent was evaporated and the product purified again by normal phase flash chromatography (SNAP Ultra 50 g, DCM/MeOH from 5% to 100% MeOH). The solvent was evaporated and the residue dried under reduced pressure. The product was obtained as a colorless solid (922 mg, 1.32 mmol, 65%, purity > 85%).

^1^H NMR (400 MHz, D_2_O) *δ*: 4.62–4.59 (m, 2H, 2 × Cys C*H*CH_2_), 3.82 (d, *J* = 4 Hz, 4H, 2 × Gly C*H*_2_COOH), 3.81–3.74 (m, 2H, 2 × Glu C*H*NH_2_), 3.12 (dd, *J* = 6 Hz, *J* = 14 Hz, 2H, Cys CHC*H*_2_S), 2.91 (dd, *J* = 8 Hz, *J* = 14 Hz, 2H, Cys CHC*H*_2_S), 2.85 (s, 8H, 2 × SC*H*_2_C*H*_2_S), 2.61–2.47 (m, 4H, 2 × Glu CH_2_C*H*_2_CONH), 2.17 (q, *J* = 8 Hz, 4H, 2 × Glu C*H*_2_CH_2_CONH) ppm.

^13^C NMR {^1^H} (100 MHz, D_2_O) *δ*: 175.8 (2 × *C*O), 174.9 (2 × *C*O), 173.9 (2 × *C*O), 172.0 (2 × *C*O), 54.1 (2 × Cys *C*HCH_2_), 53.1 (2 × Glu *C*HNH_2_), 43.1 (2 × Gly *C*H_2_COOH), 32.9 (2 × Cys CH*C*H_2_S), 31.5 & 31.4 (2 × S*C*H_2_*C*H_2_S), 31.0 (2 × Glu CH_2_*C*H_2_CONH), 26.2 (2 × Glu *C*H_2_CH_2_CONH) ppm.

HRMS (ESI/Q-TOF) *m*/*z* [M + H]^+^ calcd for C_24_H_41_N_6_O_12_S_3_ 701.1945, found: 701.1964.

FTIR (neat) 1589, 1519, 1390, 1309, 1026, 534, 522.

### *N*,*S*-Bis(*tert*-butoxycarbonyl)glutathione, *N*,*S*-Boc-GSH 11

GSH (1.23 g, 4.0 mmol, 1 equiv.) and NaHCO_3_ (1.48 g, 17.6 mmol, 4.4 equiv.) were dissolved in THF/H_2_O (28 mL, 2 : 5). Boc_2_O (3.84 g, 17.6 mmol, 4.4 equiv.) was added to the solution. The reaction mixture was stirred for 24 h at room temperature. It was then acidified to pH 2 with aq. HCl 37% and extracted with EtOAc (3 × 20 mL). The combined organic layers were dried over MgSO_4_ and the solvent was removed under reduced pressure after filtration. The crude product was obtained as a white solid (2.93 g, purity 50%). 2.02 g of the crude was purified by flash chromatography (SNAP Ultra 100 g, DCM/MeOH from 13% to 100% MeOH). After evaporation of the solvent the product was obtained as a white solid (949 mg, 1.87 mmol, 47%, purity > 85%). The product contained different stereoisomers and also mono-protected GSH as impurities.

^1^H NMR (400 MHz, DMSO-d_6_) *δ*: 12.62 (br. s., 2H, Glu & Gly COO*H*), 8.19 (d, *J* = 9 Hz, 1H, Glu N*H*), 8.08 (t, *J* = 6 Hz, 1H, Gly N*H*), 6.81 (d, *J* = 8 Hz, 1H, Cys N*H*), 4.48–4.43 (m, 1H, Cys C*H*CH_2_), 3.86–3.81 (m, 1H, Glu C*H*NH_2_), 3.64–3.62 (m, 2H, Gly C*H*_2_COOH), 3.21 (dd, *J* = 5 Hz, *J* = 14 Hz, 1H, Cys CHC*H*_2_S), 2.92 (dd, *J* = 9 Hz, *J* = 13 Hz, 1H, Cys CHC*H*_2_S), 2.18 (t, *J* = 7 Hz, 2H, Glu CH_2_C*H*_2_CONH), 1.92–1.87 (m, 1H, Glu C*H*_2_CH_2_CONH), 1.83–1.77 (m, 1H, Glu C*H*_2_CH_2_CONH), 1.44 (s, 9, C(C*H*_3_)_3_), 1.37 (s, 9H, C(C*H*_3_)_3_)) ppm.

^13^C NMR {^1^H} (100 MHz, DMSO-d_6_) *δ*: 174.6 (*C*O), 172.3 (*C*O), 171.5 (*C*O), 170.1 (*C*O), 168.6 (*C*O), 155.8 (NH*C*OOC(CH_3_)_3_), 85.3 (SCOO*C*(CH_3_)_3_), 78.3 (NHCOO*C*(CH_3_)_3_), 53.8 (Cys *C*HCH_2_), 52.4 (Glu *C*HNH_2_), 42.3 (Gly *C*H_2_COOH), 33.0 (Cys CH*C*H_2_S), 28.7 (Glu CH_2_*C*H_2_CONH), 28.2 (Glu *C*H_2_CH_2_CONH), 27.7 (NHCOOC(*C*H_3_)_3_), 21.6 (SCOOC(*C*H_3_)_3_) ppm.

HRMS (ESI/Q-TOF) *m*/*z* [M + H]^+^ calcd for C_20_H_34_N_3_O_10_S 508.1959, found: 508.1951.

FTIR (neat): 2446, 2410, 2402, 2328, 2314, 2304, 2277, 2265, 1711, 1602, 1524, 1516, 1448, 1414, 1318, 1223, 1179, 1124, 1031, 1006, 943, 907, 875, 845, 759, 735, 662, 647, 620 cm^−1^.

Mp 93 °C.

### *O*-Bis(2-(2-(hydroxy)ethylthio)ethyl)-*N*,*S*-bis(*tert*-butoxycarbonyl)glutathione, bis-*O*-HETE-*N*,*S*-Boc-GSH 12

To a solution of 11 (500 mg, 0.985 mmol, 1 equiv.) and thiodiglycol (2.65 mL, 25.61 mmol, 26 equiv.) in anhydrous EtOAc (7.5 mL) under inert gas atmosphere were added HOBt (332 mg, 2.17 mmol, 2.2 equiv.), DMAP (144 mg, 1.18 mmol, 1.2 equiv.) and EDC (0.42 mL, 2.36 mmol, 2.4 equiv.) at 0 °C. The reaction mixture was stirred for 30 min at 0 °C and then for 22 h at room temperature. It was then washed with aq. HCl (2%, 8 mL), aq. NaHCO_3_ (2%, 8 mL) and H_2_O (8 mL). The organic layer was dried over MgSO_4_ and the solvent was removed under reduced pressure. The product was obtained as a colorless viscous oil (445 mg [estimated dry mass; product still contained EtOAc (27%), which could not be removed: wet mass 622 mg], 0.62 mmol, 63%, purity > 68%).

^1^H NMR (400 MHz, CDCl_3_) *δ*: 6.99 (d, *J* = 7 Hz, 1H, Cys N*H*), 5.40 (d, *J* = 7 Hz, 1H, Glu N*H*), 4.69–4.64 (m, 1H, Cys C*H*CH_2_), 4.39–4.27 (m, 5H, SCH_2_C*H*_2_OOC-Glu & Glu C*H*NH & Gly C*H*_2_COO), 4.12 (dd, *J* = 6 Hz, *J* = 18 Hz, 1H, SCH_2_C*H*_2_OOC-Gly), 3.96 (dd, *J* = 5 Hz, *J* = 18 Hz, 1H, SCH_2_C*H*_2_OOC-Gly), 3.78–3.76 (m, 4H, 2 × C*H*_2_OH), 3.28 (dd, *J* = 5 Hz, *J* = 15 Hz, 1H, Cys CHC*H*_2_S), 3.17 (dd, *J* = 8 Hz, *J* = 15 Hz, 1H, Cys CHC*H*_2_S), 2.99 (br. s, 1H, O*H*), 2.84–2.76 (m, 9H, 2 × HOCH_2_C*H*_2_SC*H*_2_CH_2_OOC & O*H*), 2.43–2.31 (m, 2H, Glu CH_2_C*H*_2_CONH), 2.20–2.15 (m, 1H, Glu C*H*_2_CH_2_CONH), 2.09–2.04 (m, 1H, Glu C*H*_2_CH_2_CONH), 1.50 (s, 9H, C(C*H*_3_)_3_), 1.44 (s, 9H, C(C*H*_3_)_3_) ppm.

^13^C NMR {^1^H} (100 MHz, CDCl_3_) *δ*: 172.8 (*C*O), 172.3 (*C*O), 170.5 (*C*O), 170.0 (*C*O), 169.4 (*C*O), 85.9 (SCOO*C*(CH_3_)_3_), 77.2 (NHCOO*C*(CH_3_)_3_), 64.7 (SCH_2_*C*H_2_OOC), 64.6 (SCH_2_*C*H_2_OOC), 61.0 (*C*H_2_OH), 61.0 (*C*H_2_OH), 53.8 (Cys *C*HCH_2_), 52.8 (Glu *C*HNH), 41.5 (Gly *C*H_2_COO), 35.4 (*C*H_2_CH_2_OH), 35.4 (*C*H_2_CH_2_OH), 32.0 (Cys CH*C*H_2_S), 30.5 (S*C*H_2_CH_2_OOC), 30.3 (S*C*H_2_CH_2_OOC), 28.3 (C(*C*H_3_)_3_), 28.2 (C(*C*H_3_)_3_) ppm.

HRMS (ESI/Q-TOF) *m*/*z* [M + H]^+^ calcd for C_28_H_50_N_3_O_12_S_3_ 716.2551, found: 716.2557.

FTIR (neat): 3099, 3001, 2987, 2955, 2939, 2882, 2224, 2196, 21 886, 2157, 2134, 2118, 1960, 1937, 1741, 1697, 1655, 1507, 1476, 1456, 1394, 1369, 1349, 1285, 1210, 1162, 1123, 1058, 1032, 1006, 963, 895, 855, 834, 651, 568 cm^−1^.

### *O*-Bis(2-(2-(hydroxy)ethylthio)ethyl)glutathione, bis-*O*-HETE–GSH 8

A solution of TFA : TFE : H_2_O (8.65 mL, 80 : 10 : 10) was added dropwise to 12 (376 mg, 0.525 mmol, 1 equiv.) at 0 °C. After addition, the mixture was allowed to warm to room temperature and was stirred for 2 h. The solvent was evaporated under reduced pressure. The crude product was purified by flash chromatography (SNAP Ultra 50 g, DCM/MeOH from 2% to 100% MeOH) to afford a colorless viscous oil (328 mg, 0.636 mmol, 91%, purity > 75%).

^1^H NMR (400 MHz, D_2_O) *δ*: 4.57 (t, *J* = 7 Hz, 1H, Cys C*H*CH_2_), 4.46 (t, *J* = 6 Hz, 2H, SCH_2_C*H*_2_OOC-Glu), 4.36 (t, *J* = 6 Hz, 2H, Gly C*H*_2_COO), 4.23 (t, *J* = 7 Hz, 1H, Glu C*H*NH), 4.08 (d, *J* = 5 Hz, 2H, SCH_2_C*H*_2_OOC-Gly), 3.77 (td, *J* = 2 Hz, *J* = 8 Hz, 4H, 2 × C*H*_2_OH), 2.95 (q, *J* = 7 Hz, 4H, 2 × SC*H*_2_CH_2_OOC), 2.89 (t, *J* = 7 Hz, 2H, Cys CHC*H*_2_S), 2.78 (td, *J* = 2 Hz, *J* = 8 Hz, 4H, 2 × SC*H*_2_CH_2_OH), 2.72–2.58 (m, 2H, Glu CH_2_C*H*_2_CONH), 2.35–2.23 (m, 2H, Glu C*H*_2_CH_2_CONH) ppm.

^13^C NMR {^1^H} (100 MHz, D_2_O) *δ*: 174.1 (*C*O), 172.7 (*C*O), 171.0 (*C*O), 169.5 (*C*O), 65.3 (SCH_2_*C*H_2_OOC), 64.7 (SCH_2_*C*H_2_OOC), 60.3 (2 × *C*H_2_OH), 55.5 (Cys *C*HCH_2_), 52.1 (Glu *C*HNH_2_), 41.3 (Gly *C*H_2_COO), 33.6 (S*C*H_2_CH_2_OH), 33.5 (S*C*H_2_CH_2_OH), 30.6 (Glu CH_2_*C*H_2_CONH), 29.7 (S*C*H_2_CH_2_OOC), 29.6 (S*C*H_2_CH_2_OOC), 25.4 (Glu *C*H_2_CH_2_CONH), 25.3 (Cys CH*C*H_2_S) ppm.

HRMS (ESI/Q-TOF) *m*/*z* [M + H]^+^ calcd for C_18_H_34_N_3_O_8_S_3_ 516.1508, found: 516.1503.

FTIR (neat): 2264, 2248, 2237, 2228, 2044, 1744, 1666, 1527, 1462, 1423, 1360, 1196, 1131, 1065, 722, 630 cm^−1^.

### *O*-(2-(2-(*tert*-Butoxy)ethylthio)ethyl)-*N*-(*tert*-butoxycarbonyl)-l-glutamic acid 5-((9*H*-fluoren-9-yl)methyl) ester, Boc-Glu(OFm)-*O*-ETEO*t*Bu 14

A solution of DIPEA (421 μL, 2.4 mmol, 1 equiv.) in degassed DCM (15 mL) was added to Boc-Glu(OFm)–OH (1000 g, 2.4 mmol, 1 equiv.). The solution was stirred 5 min and a solution of HCTU (1458 mg, 3.5 mmol, 1.5 equiv.) and DIPEA (421 μL, 2.4 mmol, 1 equiv.) in degassed DCM/DFM (40 mL, 1 : 1) was added dropwise at 0 °C. The solution was stirred 10 min at 0 °C and a solution of 1 (838 mg, 4.7 mmol, 2 equiv.) in DCM (5 mL) was then added. The reaction mixture was stirred for 1 h at 0 °C and 24 h at room temperature. The solution was washed with aq HCl (2%, 20 mL), aq. NaHCO_3_ (2%, 20 mL) and H_2_O (20 mL). The organic fraction was dried over Na_2_SO_4_, the solvent was concentrated under reduced pressure and the crude product was purified by normal phase flash chromatography (SNAP Ultra 25 g, hexane/EtOAc from 0% to 20% EtOAc). The product was obtained as a yellowish oil (1.5 g, 110%, purity > 66%).

^1^H NMR (400 MHz, CDCl_3_) *δ*: 7.77 (d, *J* = 8 Hz, 2H, Fm), 7.59 (d, *J* = 7 Hz, 2H, Fm), 7.42 (t, *J* = 7 Hz, 2H, Fm), 7.31 (t, *J* = 7 Hz, 2H, Fm), 5.15 (d, *J* = 8 Hz, 1H, N*H*), 4.39 (d, *J* = 7 Hz, 2H, Fm C*H*_2_), 4.32 (t, *J* = 7 Hz, 2H, Fm C*H* & C(O)C*H*), 4.21 (t, *J* = 7 Hz, 2H,COOC*H*_2_CH_2_S), 3.76 (t, *J* = 6 Hz, 2H, C*H*_2_O*t*Bu), 3.53 (t, *J* = 7 Hz, 2H), 2.83 (t, *J* = 7 Hz, 2H, COOCH_2_C*H*_2_S), 2.67 (t, *J* = 7 Hz, 2H, SC*H*_2_CH_2_O*t*Bu), 2.52–2.48 (m, 2H, C*H*_2_CH_2_CH), 2.23–1.92 (m, 2H, CH_2_C*H*_2_CH), 1.62 (s, 9H, C(C*H*_3_)_3_), 1.17 (s, 9H, C(C*H*_3_)_3_) ppm.

^13^C NMR {^1^H} (100 MHz, CDCl_3_) *δ*: 172.7 (*C*O), 172.0 (*C*O), 156.6 (*C*OO*t*Bu), 143.8 (Fm), 141.3 (Fm), 127.8 (Fm), 127.15 (Fm), 125.0 (Fm), 120.05 (Fm), 73.6 (*C*(CH_3_)_3_), 73.3 (*C*(CH_3_)_3_), 66.4 (Fm *C*H_2_), 64.5 (*C*H_2_COO*t*Bu), 62.1 (COO*C*H_2_CH_2_S), 52.9 (NH*C*H), 46.8 (Fm *C*H), 36.5 (S*C*H_2_CH_2_O*t*Bu), 32.9 (COOCH_2_*C*H_2_S), 30.3 (*C*H_2_COOFm), 27.5 (*C*H_2_CH_2_COOFm), 27.5 (*C*H_3_) ppm.

HRMS (ESI/Q-TOF) *m*/*z* [M + H]^+^ calcd for C_32_H_44_NO_7_S 586.2833, found: 586.2824.

FTIR (neat): 3376, 2973, 2930, 1737, 1714, 1504, 1477, 1450, 1390, 1364, 1294, 1251, 1162, 1091, 1071, 1024, 989, 909, 883, 760, 741, 631, 532 cm^−1^.

### *O*-(2-(2-(*tert*-Butoxy)ethylthio)ethyl)-*N*-(*tert*-butoxycarbonyl)-l-glutamic acid, Boc-Glu(OH)-*O*-ETEO*t*Bu 15

17 (200 mg, 0.3 mmol, 1 equiv.) was dissolved in 20% piperidine in DMF (6 mL) and the solution was stirred at room temperature for 24 h. The reaction progress was monitored by direct injection (negative ESI mode) into the Dalton mass detector. The solvent was removed under reduced pressure and the crude product was purified by normal phase flash chromatography (SNAP Ultra 10 g, hexane/EtOAc from 0% to 80% EtOAc) and dried by lyophilisation. The product was obtained as a colorless oil (90 mg, 0.2 mmol, 65%, purity > 97%).

^1^H NMR (400 MHz, CD_3_CN) *δ*: 7.49 (d, *J* = 7.5 Hz, 1H, N*H*), 4.27 (m, 2H, CHCOOC*H*_2_), 4.17 (m, 1H, NHC*H*), 3.55 (t, *J* = 6.3 Hz, 2H, C*H*_2_O*t*Bu), 2.82 (t, *J* = 6.6 Hz, 2H, COOC*H*_2_CH_2_S), 2.67 (t, *J* = 6.5 Hz, 2H, C*H*_2_CH_2_O*t*Bu), 2.41 (t, *J* = 7.2 Hz, 2H, CH_2_C*H*_2_COOH), 2.09–1.86 (m, 2H, C*H*_2_CH_2_COOH), 1.43 (s, 9H, C(C*H*_3_)_3_), 1.19 (s, 9H, C(C*H*_3_)_3_) ppm.

^13^C NMR {^1^H} (100 MHz, CD_3_CN) *δ*: 174.2 (*C*O), 172.7 (*C*O), 156.2 (*C*OO*t*Bu), 79.6 (*C*(CH_3_)_3_), 73.3 (*C*(CH_3_)_3_), 64.8 (*C*H_2_COO*t*Bu), 62.4 (COO*C*H_2_CH_2_S), 53.6 (NH*C*H), 33.1, 30.0 (S*C*H_2_CH_2_O*t*Bu), 28.1 (COOCH_2_*C*H_2_S), 27.4 (*C*H_2_COOH), 27.2 (*C*H_3_), 27.1 (*C*H_2_CH_2_COOH) ppm.

HRMS (ESI/Q-TOF) *m*/*z* [M + H]^+^ calcd for C_9_H_18_NO_4_ 408.2051, found: 408.2045.

FTIR (neat): 3343, 2974, 2933, 1710, 1598, 1566, 1521, 1494, 1468, 1455, 1421, 1391, 1365, 1295, 1252, 1161, 1069, 1055, 1029, 1013, 992, 967, 913, 883, 858, 780, 756, 741, 727, 694, 677, 645, 546 cm^−1^.

### *O*-(2-(2-(Hydroxy)ethylthio)ethyl)glutathione, *O*-HETE–GSH 9

The synthesis of *O*-HETE–GSH 9 was performed by SPPS on a 0.2 mmol scale. The preloaded H-Gly-2-ClTrt resin (182 mg, loading 1.1 mmol g^−1^) was swelled with DMF (4.5 mL) over 2 h at room temperature. To the resin was then added Fmoc-Cys(Trt)–OH (498 mg, 0.8 mmol, 4 equiv.) in DMF (1.29 mL), DIC (128 μL, 0.8 mmol, 4 equiv.) in DMF (1.52 mL) and Oxyma pure (117 mg, 0.8 mmol, 4 equiv.) in DMF (1.65 mL). The reaction mixture was stirred for 3 h at room temperature. After DMF washes, a solution of piperidine 20% in DMF (4.5 mL, 90 equiv.) was added twice, for 5 min and 15 min agitation respectively.

After washing steps with DMF, Boc-Glu(OH)-OETEO*t*Bu 18 (346 mg, 0.8 mmol, 4 equiv.) in DMF (1.42 mL), DIC (128 μL, 0.8 mmol, 4 equiv.) in DMF (1.52 mL) and Oxyma pure (117 mg, 0.8 mmol, 4 equiv.) in DMF (1.65 mL) were added to the resin. The reaction mixture was stirred for 3 h at room temperature, followed by a pre-cleavage wash of the resin with DCM (4 × 9 mL). An additional pre-cleavage wash with DCM (4.5 mL), MeOH (4.5 mL) and DCM (4.5 mL) was performed by the instrument. The resin was transferred into a new flask and the following cleavage cocktail was added: TFA : TIS : H_2_O (10 mL, 95 : 2.5 : 2.5). The reaction mixture was stirred for 2 h at room temperature and the resin was then filtered and washed with TFA. The solution was concentrated under reduced pressure and the crude product was precipitated in ice cold Et_2_O. The final purification was performed by reversed phase flash chromatography (SNAP Ultra C18 12 g, H_2_O/MeCN from 0% to 60% MeCN). The product was obtained as a white solid (63.5 mg, 77%, purity > 90%).

^1^H NMR (400 MHz, D_2_O) *δ*: 4.48 (m, 1H, NH_2_C*H*), 4.36 (t, *J* = 6.1 Hz, 2H, CHCOOC*H*_2_), 4.14 (t, *J* = 6.1 Hz, 1H, NHC*H*), 3.79 (s, 2H), 3.67 (t, *J* = 6.2 Hz, 2H, C*H*_2_OH), 2.88–2.83 (m, 4H, S(C*H*_2_)_2_), 2.69 (t, *J* = 6.2 Hz, 2H), 2.57 (m, 2H, CHCH_2_C*H*_2_), 2.19 (m, 2H, CHC*H*_2_) ppm.

^13^C NMR {^1^H} (100 MHz, D_2_O) *δ*: 174.8 (*C*O), 174.2 (*C*O), 171.8, 169.5, 65.3 (CHCOO*C*H_2_), 60.3 (*C*H_2_OH), 55.5 (*C*HN), 52.1, 42.4, 33.5 (*C*H_2_COOH), 30.7 (S*C*H_2_CH_2_OH), 29.6 (COOCH_2_*C*H_2_S), 25.4 (*C*H_2_CH_2_COOH), 25.3 ppm.

HRMS (ESI/Q-TOF) *m*/*z* [M + H]^+^ calcd for C_14_H_26_N_3_O_7_S_2_ 412.1206, found: 412.1209.

IR (neat): 3730, 3627, 2362, 2338, 1017 cm^−1^.

## Conflicts of interest

The authors declare no competing financial interests.

## Supplementary Material

RA-008-C8RA03360A-s001
