# Supplementary material for: Synthesis of different glutathione–sulfur mustard adducts of verified and potential biomarkers
Source: RSC Adv. 2018 Jun 29;8(42):23881–90. doi: 10.1039/c8ra03360a (PMC9081735; doi:10.1039/c8ra03360a)
Supplement: RA-008-C8RA03360A-s001 [file RA-008-C8RA03360A-s001.pdf]

Electronic Supplementary Material (ESI) for RSC Advances.
This journal is © The Royal Society of Chemistry 2018

SUPPORTING INFORMATION

Synthesis of Glutathione-Sulfur Mustard Bioadducts for the Verification of Potential Biomarkers

Andreas Bielmann[a][b], Nicolas Sambiagio[a][c], Nathalie Wehr[a][b], Sandrine Gerber[c], Christian G. Bochet[b], Christophe Curty*[a]

[a]Spiez Laboratory, Austrasse, 3700 Spiez, Switzerland
[b]University of Fribourg, Departement of Chemistry, Chemin du Musée 9, 1700 Fribourg, Switzerland
[c]Ecole Polytechnique Fédéral de Lausanne, Institute of Chemical Sciences and Engineering, Station 6, 1015 Lausanne, Switzerland

christophe.curty@babs.admin.ch

Table of Contents

^{1}H NMR and ^{13}C NMR of **1** S2

^{1}H NMR and ^{13}C NMR of **2** S4

^{1}H NMR and ^{13}C NMR of **3** S6

^{1}H NMR and ^{13}C NMR of **4** by alkylation of GSH S7

^{1}H NMR and ^{13}C NMR of **5** S10

^{1}H NMR and ^{13}C NMR of **6** S12

^{1}H NMR and ^{13}C NMR of **4** by SPPS S14

^{1}H NMR and ^{13}C NMR of **7** S16

^{1}H NMR and ^{13}C NMR of **11** S18

^{1}H NMR and ^{13}C NMR of **12** S20

^{1}H NMR and ^{13}C NMR of **8** S22

^{1}H NMR and ^{13}C NMR of **14** S24

^{1}H NMR and ^{13}C NMR of **15** S26

^{1}H NMR and ^{13}C NMR of **9** S28

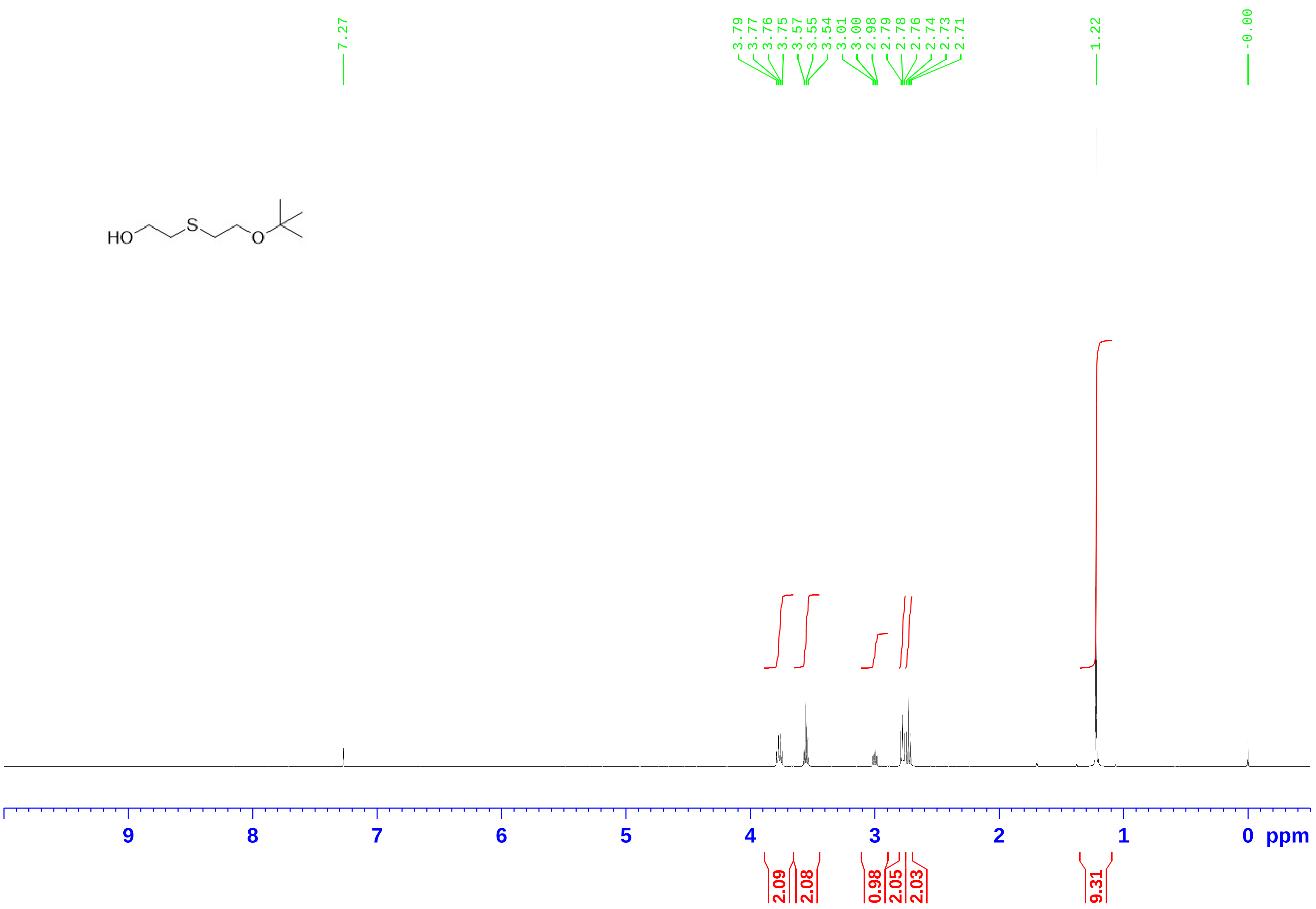

HO
S
O
7.27
3.79
3.77
3.76
3.75
3.57
3.55
3.54
3.01
3.00
2.98
2.79
2.78
2.76
2.74
2.73
2.71
1.22
-0.00
9
8
7
6
5
4
3
2
1
0
ppm
2.09
2.08
0.98
2.05
2.03
9.31

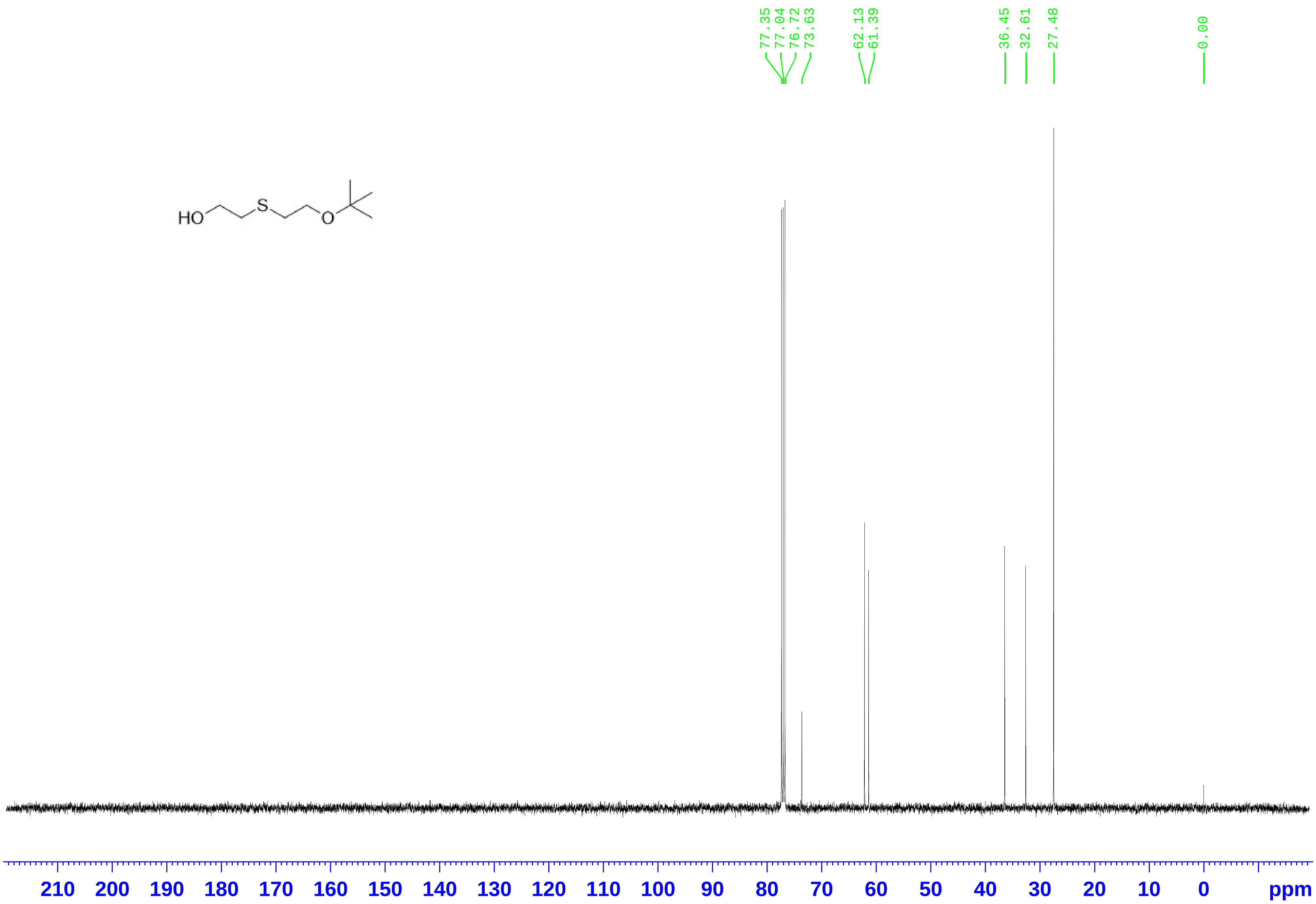
77.35
77.04
76.72
73.63
62.13
61.39
36.45
32.61
27.48
0.00
HO
S
O
210
200
190
180
170
160
150
140
130
120
110
100
90
80
70
60
50
40
30
20
10
0
ppm

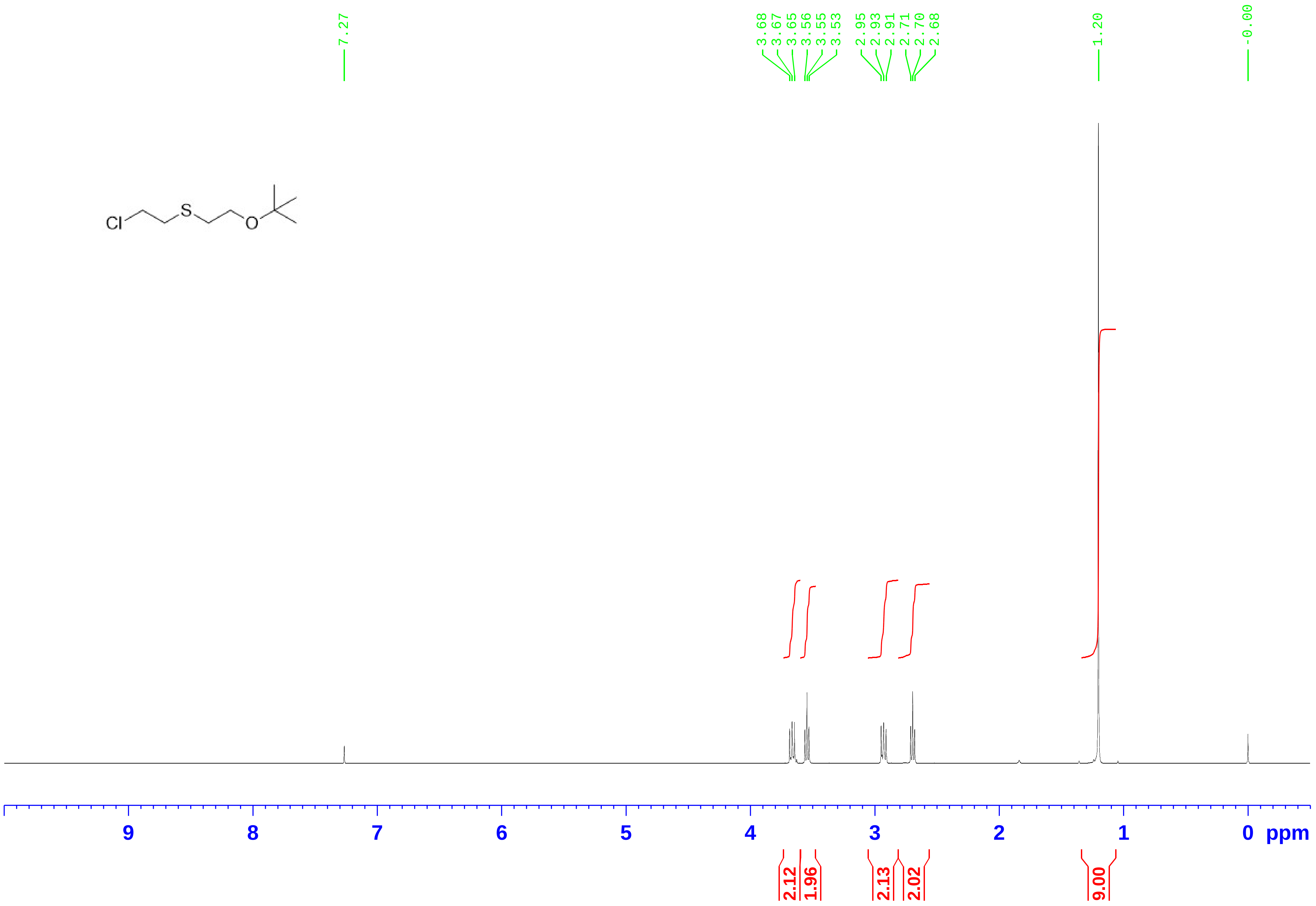

7.27
3.68
3.67
3.65
3.56
3.55
3.53
2.95
2.93
2.91
2.71
2.70
2.68
1.20
-0.00
Cl
S
O
9
8
7
6
5
4
3
2
1
0
ppm
2.12
1.96
2.13
2.02
9.00

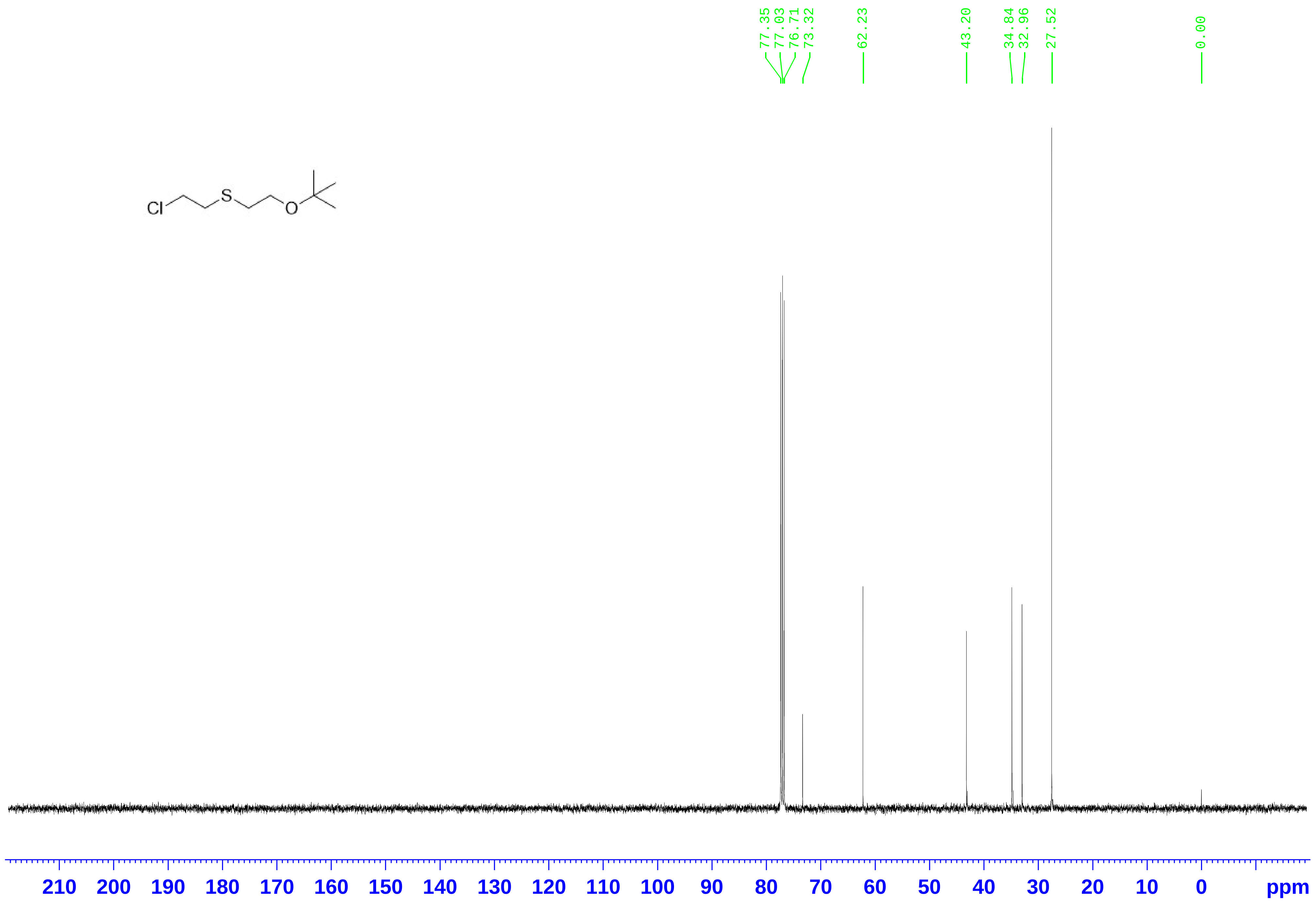

Cl
S
O
77.35
77.03
76.71
73.32
62.23
43.20
34.84
32.96
27.52
0.00
210 200 190 180 170 160 150 140 130 120 110 100 90 80 70 60 50 40 30 20 10 0 ppm

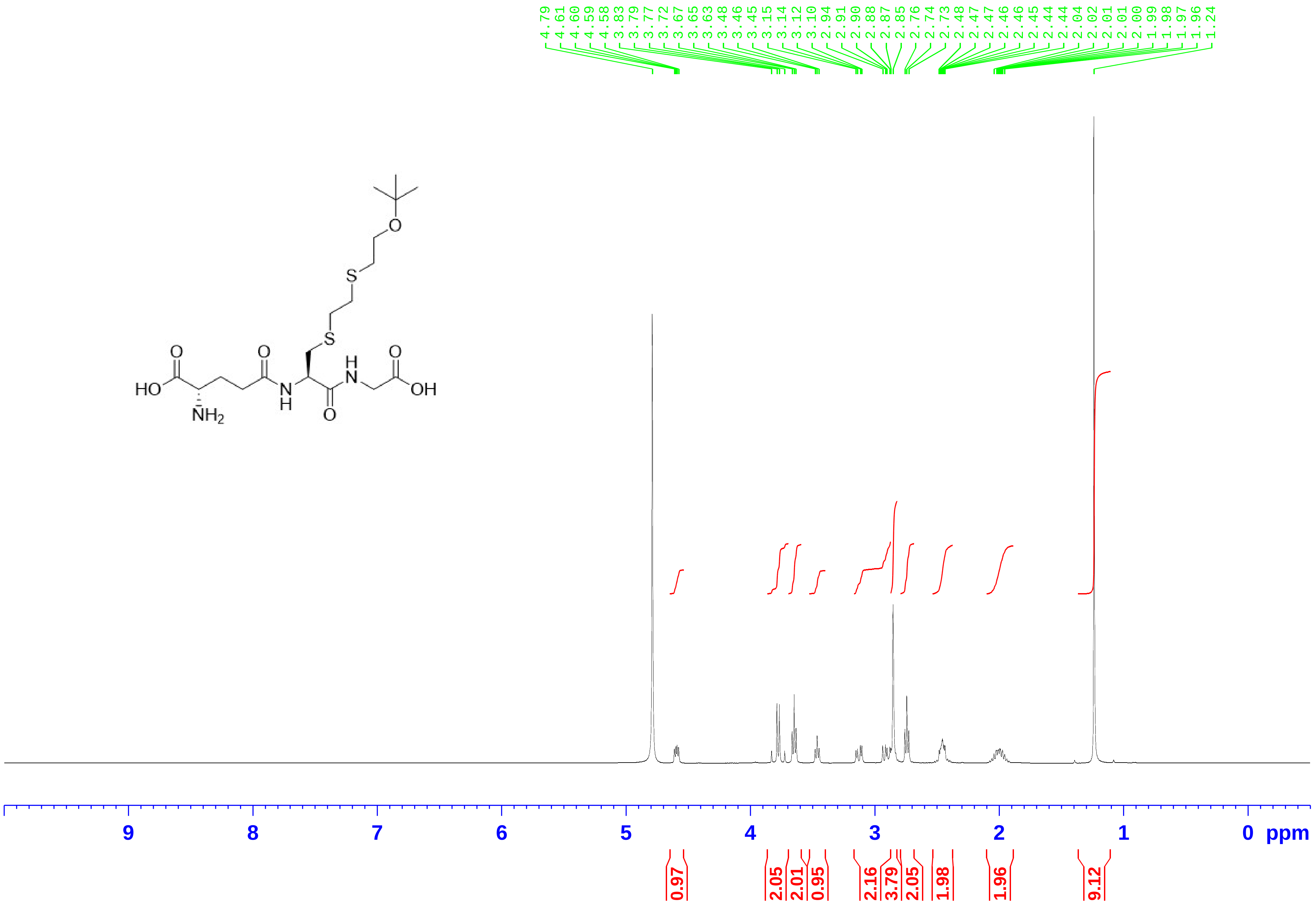

4.79
4.61
4.60
4.59
4.58
3.83
3.79
3.77
3.72
3.67
3.65
3.63
3.48
3.46
3.45
3.15
3.14
3.12
3.10
2.94
2.91
2.90
2.88
2.87
2.85
2.76
2.74
2.73
2.48
2.47
2.47
2.46
2.46
2.45
2.44
2.44
2.04
2.02
2.01
2.01
2.00
1.99
1.98
1.97
1.96
1.24
HO
O
NH2
N
H
S
S
O
H
N
OH
9
8
7
6
5
4
3
2
1
0
ppm
0.97
2.05
2.01
0.95
2.16
3.79
2.05
1.98
1.96
9.12

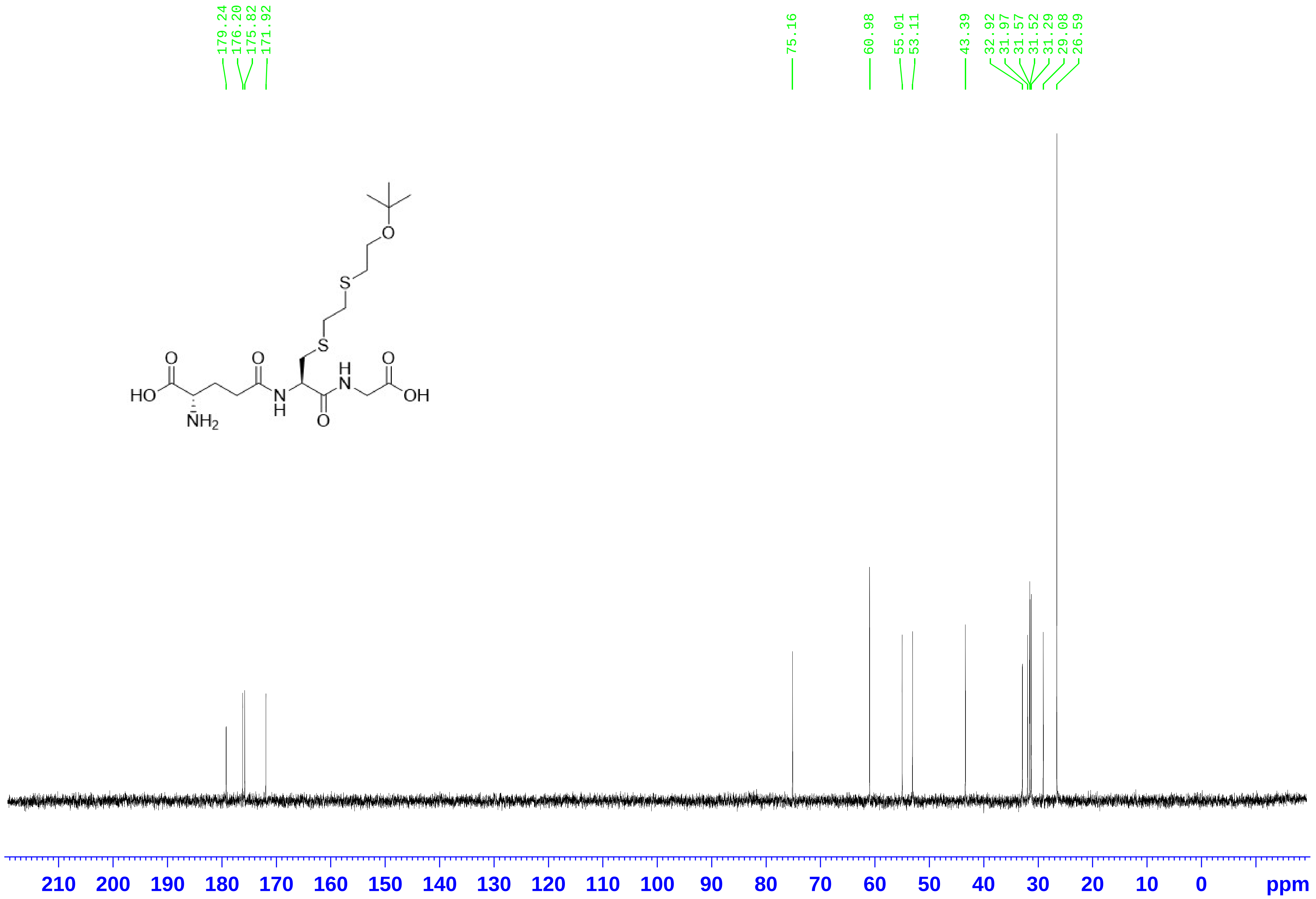
179.24
176.20
175.82
171.92
75.16
60.98
55.01
53.11
43.39
32.92
31.97
31.57
31.52
31.29
29.08
26.59
HO
O
NH2
N
H
S
O
OH
210
200
190
180
170
160
150
140
130
120
110
100
90
80
70
60
50
40
30
20
10
0
ppm

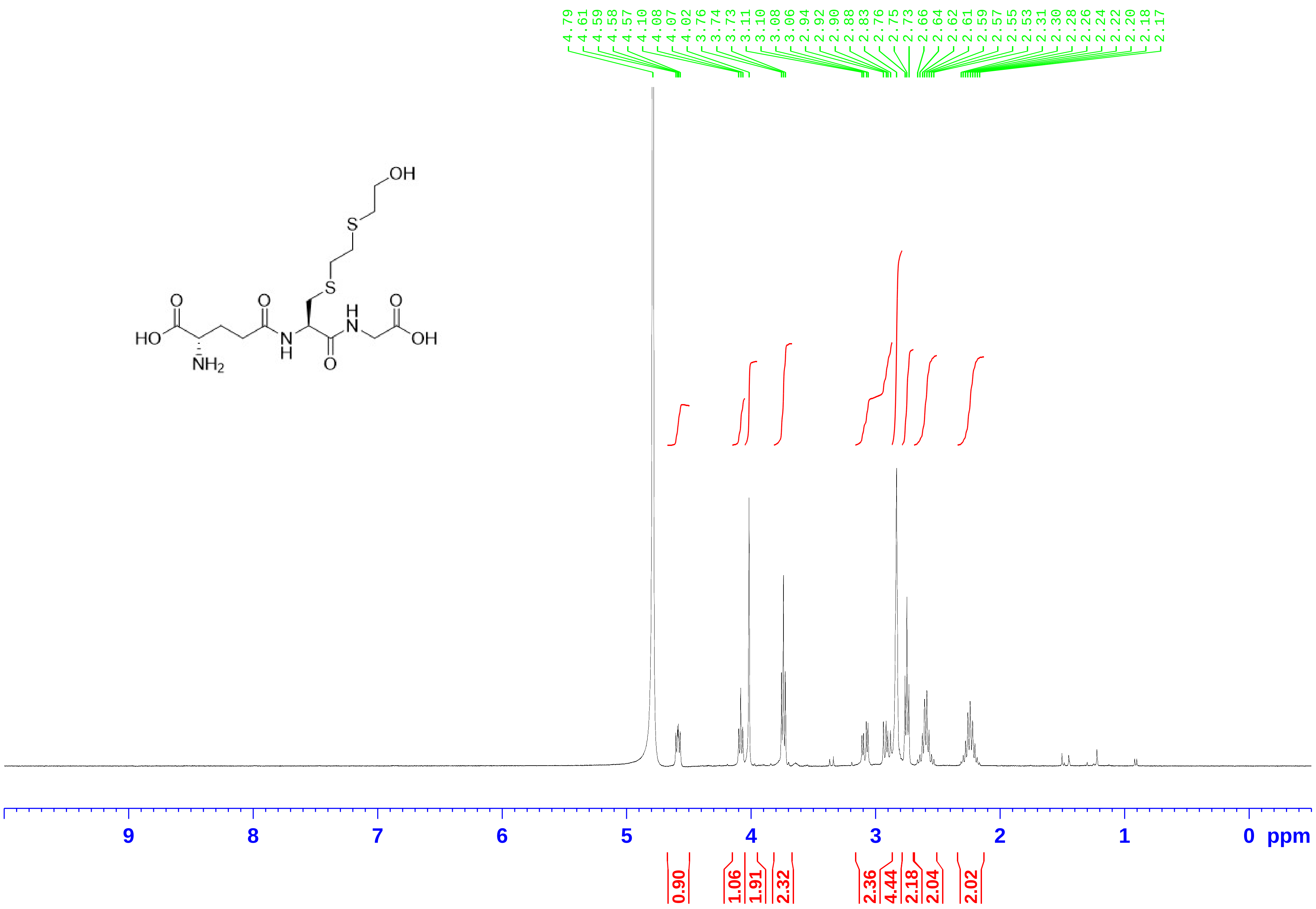
4.79
4.61
4.59
4.58
4.57
4.10
4.08
4.07
4.02
3.76
3.74
3.73
3.11
3.10
3.08
3.06
2.94
2.92
2.90
2.88
2.83
2.76
2.75
2.73
2.66
2.64
2.62
2.61
2.59
2.57
2.55
2.53
2.31
2.30
2.28
2.26
2.24
2.22
2.20
2.18
2.17
HO
O
NH2
N
H
O
S
S
OH
H
N
O
O
OH
9
8
7
6
5
4
3
2
1
0
ppm
0.90
1.06
1.91
2.32
2.36
4.44
2.18
2.04
2.02

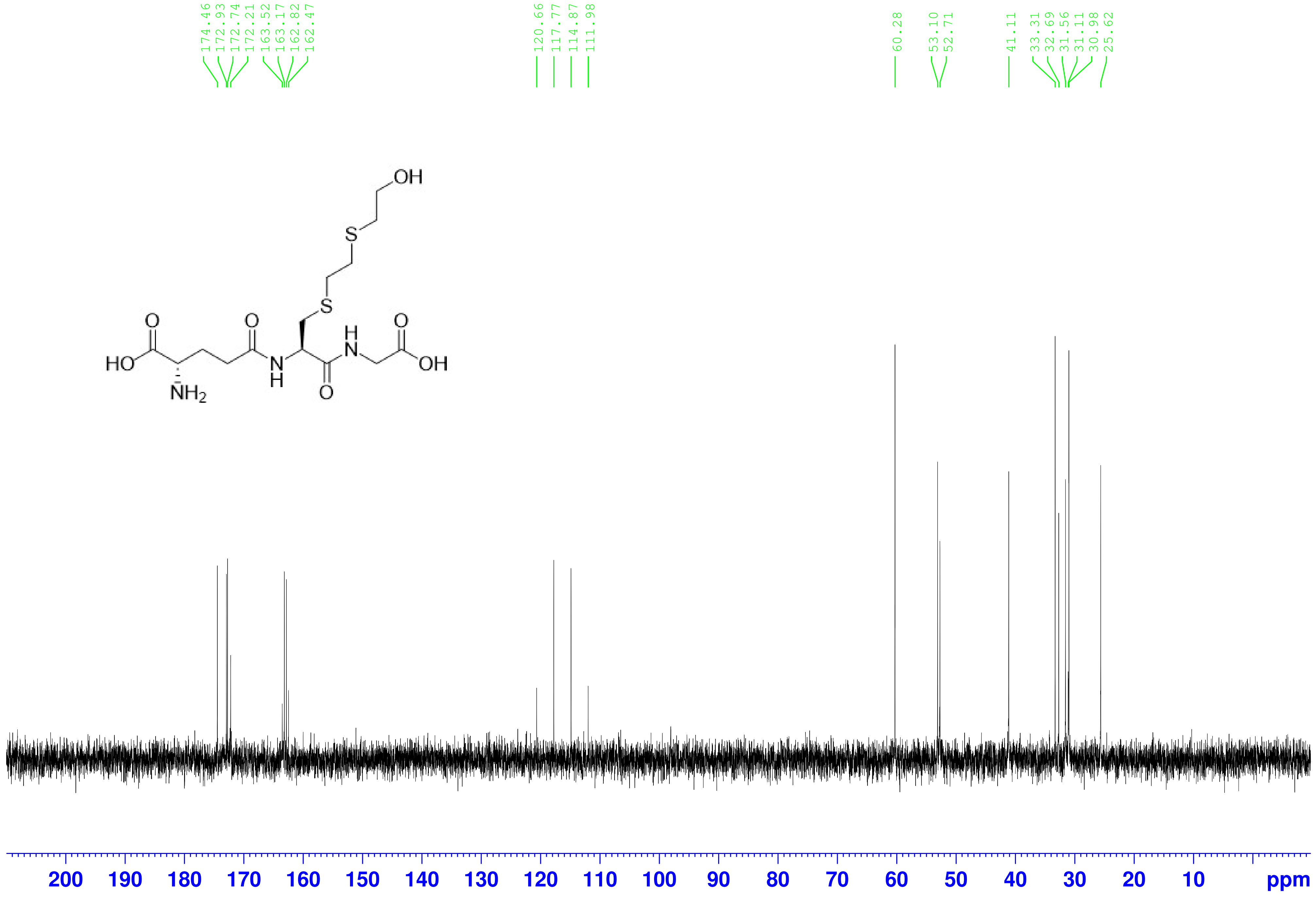
174.46
172.93
172.74
172.21
163.52
163.17
162.82
162.47
120.66
117.77
114.87
111.98
60.28
53.10
52.71
41.11
33.31
32.69
31.56
31.11
30.98
25.62
HO
O
NH2
O
N
H
S
S
OH
H
N
O
O
OH
200
190
180
170
160
150
140
130
120
110
100
90
80
70
60
50
40
30
20
10
ppm

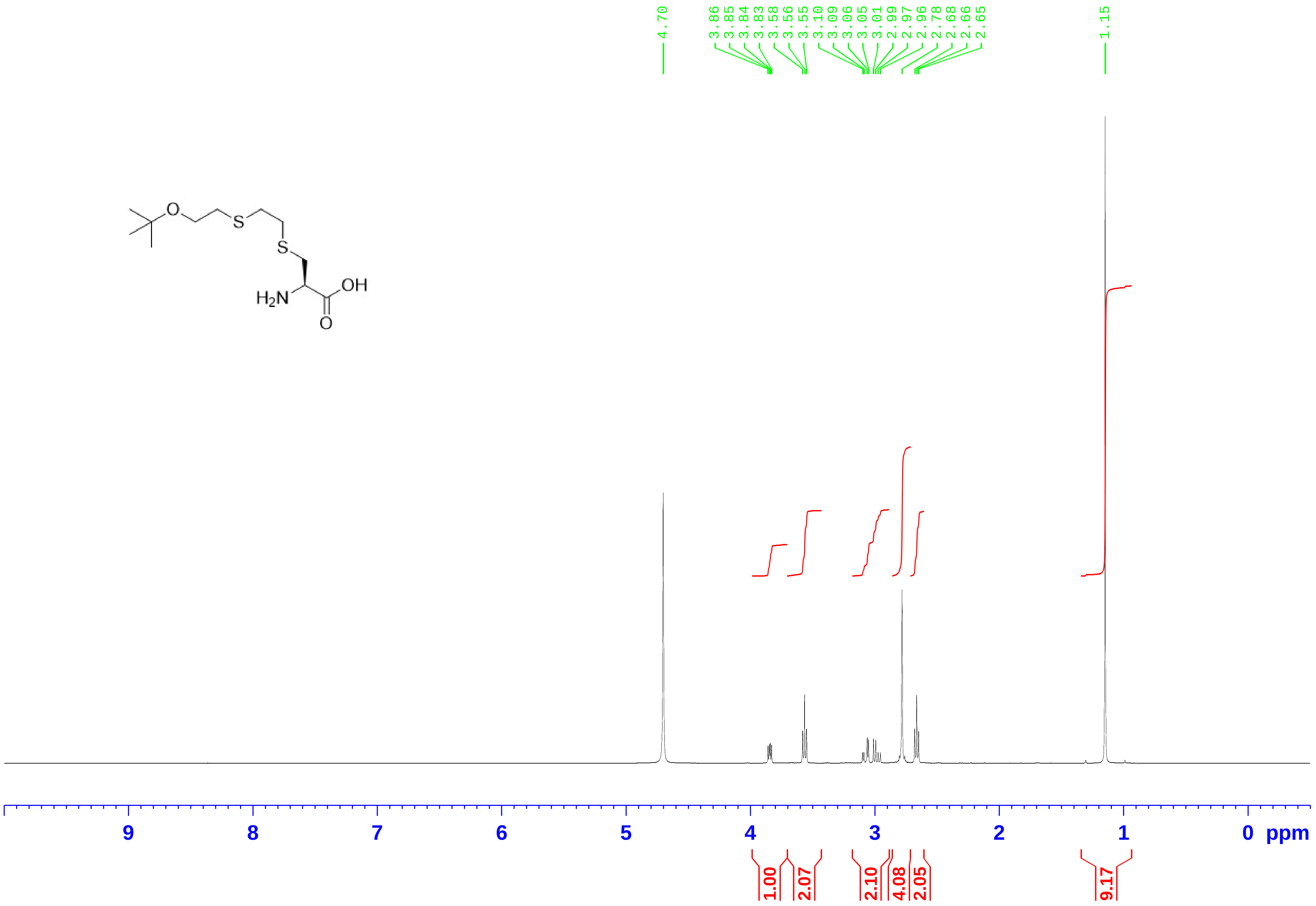

4.70
3.86 3.85 3.84 3.83 3.58 3.56 3.55 3.10 3.09 3.06 3.05 3.01 2.99 2.97 2.96 2.78 2.68 2.66 2.65
1.15
H2N
OH
O
S
S
O
9
8
7
6
5
4
3
2
1
0
ppm
1.00
2.07
2.10
4.08
2.05
9.17

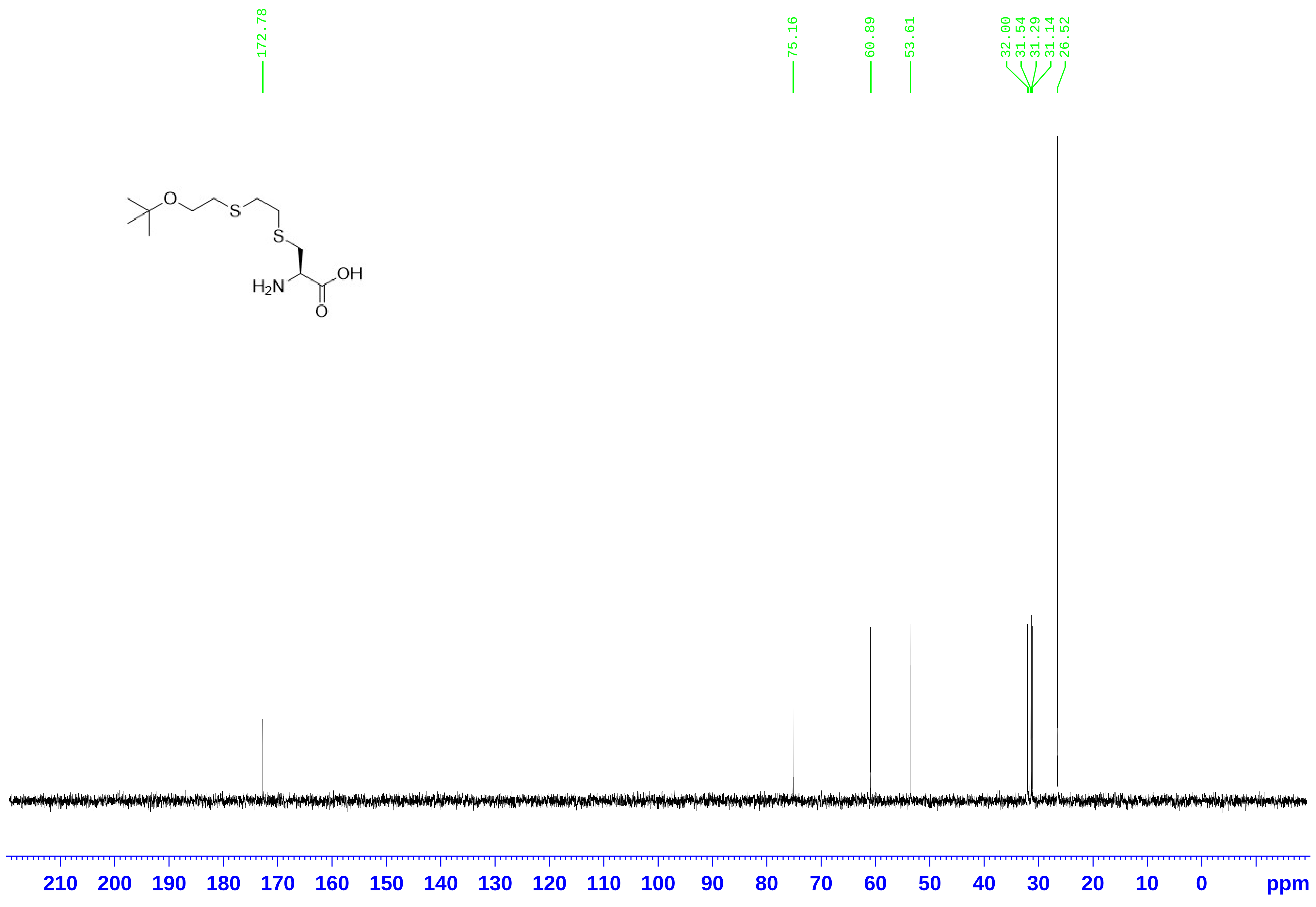

172.78
75.16
60.89
53.61
32.00
31.54
31.29
31.14
26.52
OH
H_2N
O
S
S
O
210 200 190 180 170 160 150 140 130 120 110 100 90 80 70 60 50 40 30 20 10 0 ppm

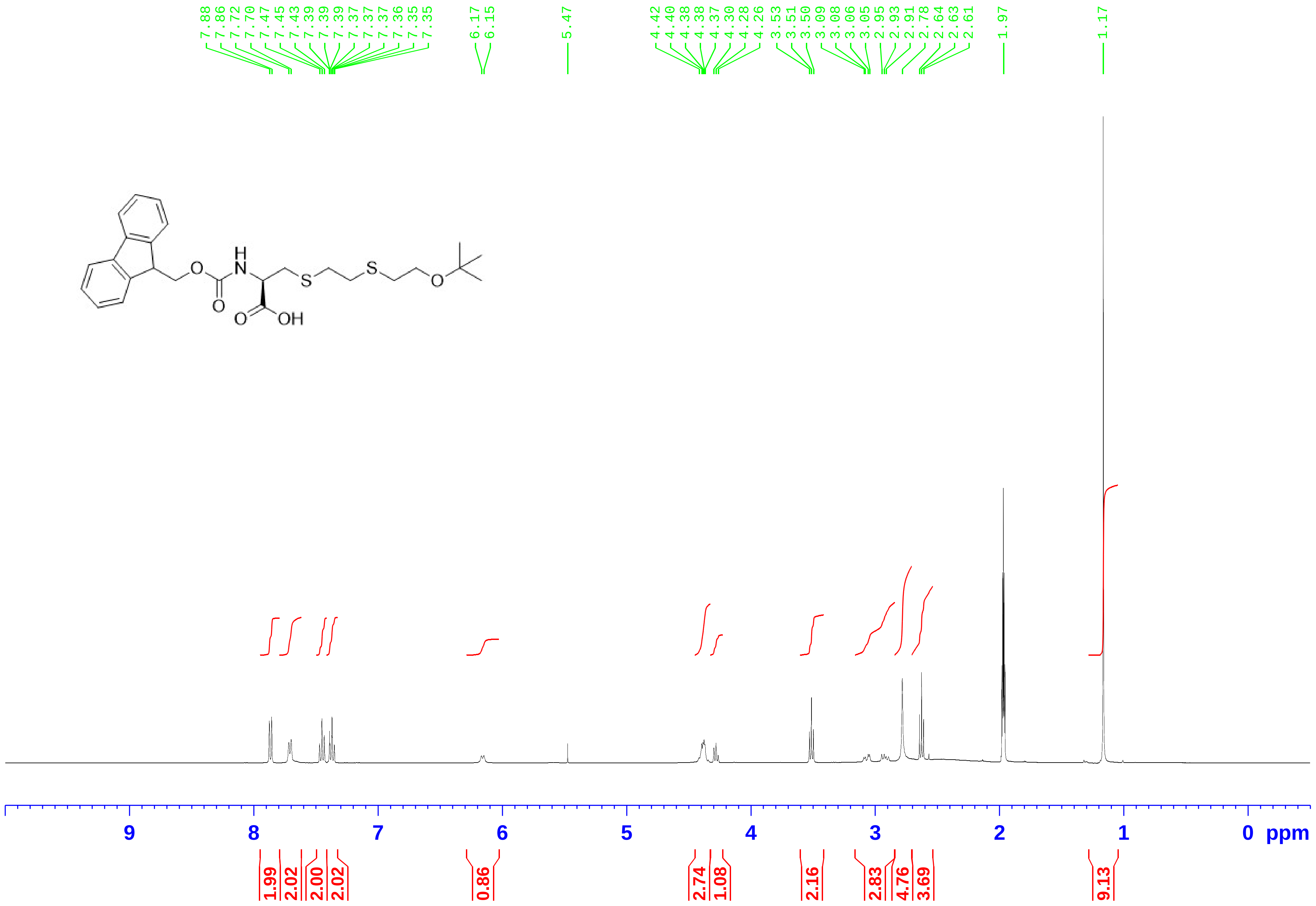

7.88
7.86
7.72
7.70
7.47
7.45
7.43
7.39
7.39
7.39
7.37
7.37
7.37
7.36
7.35
7.35
6.17
6.15
5.47
4.42
4.40
4.38
4.38
4.37
4.30
4.28
4.26
3.53
3.51
3.50
3.09
3.08
3.06
3.05
2.95
2.93
2.91
2.78
2.64
2.63
2.61
1.97
1.17
H
N
O
O
O
OH
S
S
O
9
8
7
6
5
4
3
2
1
0
ppm
1.99
2.02
2.00
2.02
0.86
2.74
1.08
2.16
2.83
4.76
3.69
9.13

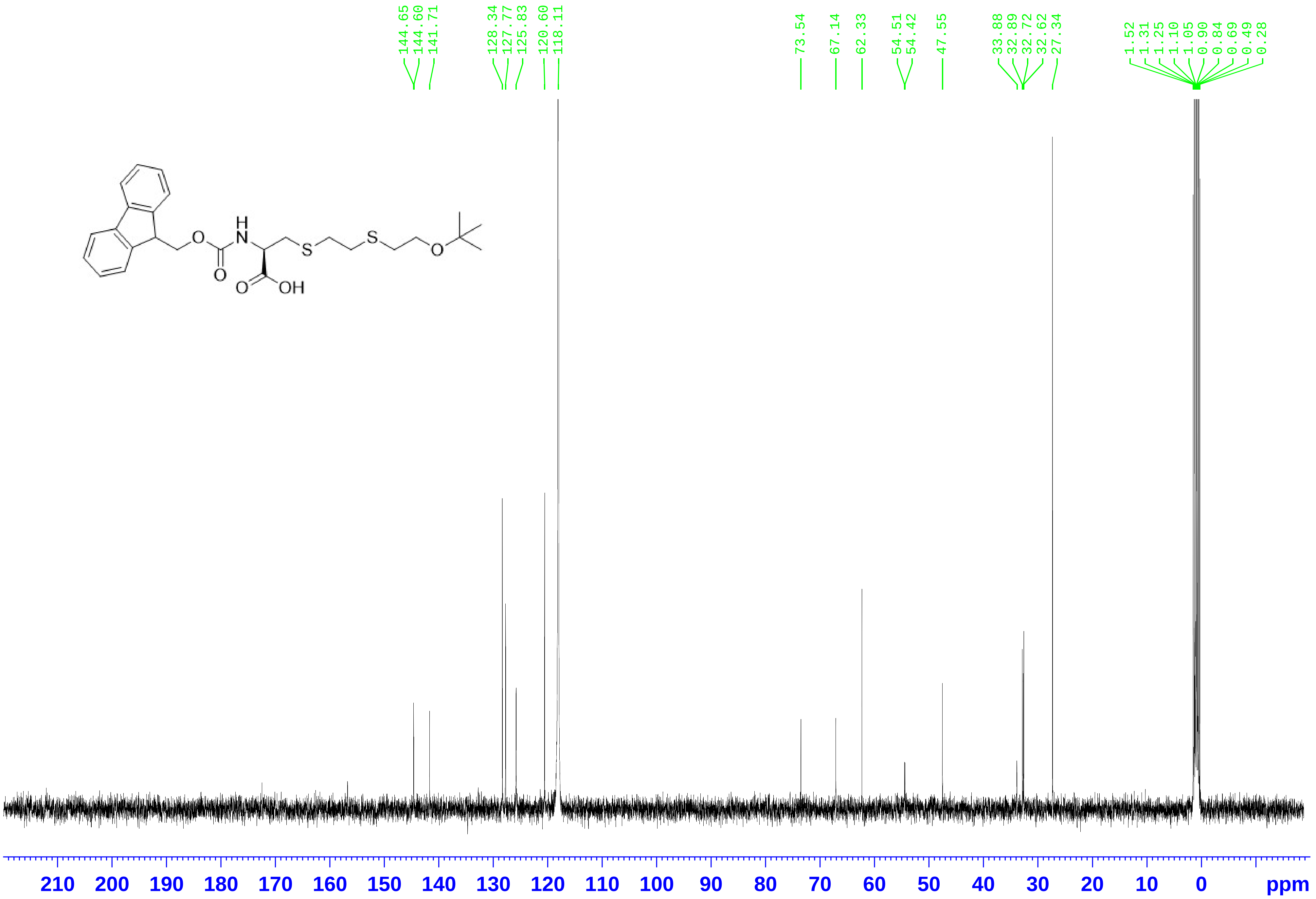

144.65
144.60
141.71
128.34
127.77
125.83
120.60
118.11
73.54
67.14
62.33
54.51
54.42
47.55
33.88
32.89
32.72
32.62
27.34
1.52
1.31
1.25
1.10
1.05
0.90
0.84
0.69
0.49
0.28
H
N
O
O
S
S
O
O
OH
210
200
190
180
170
160
150
140
130
120
110
100
90
80
70
60
50
40
30
20
10
0
ppm

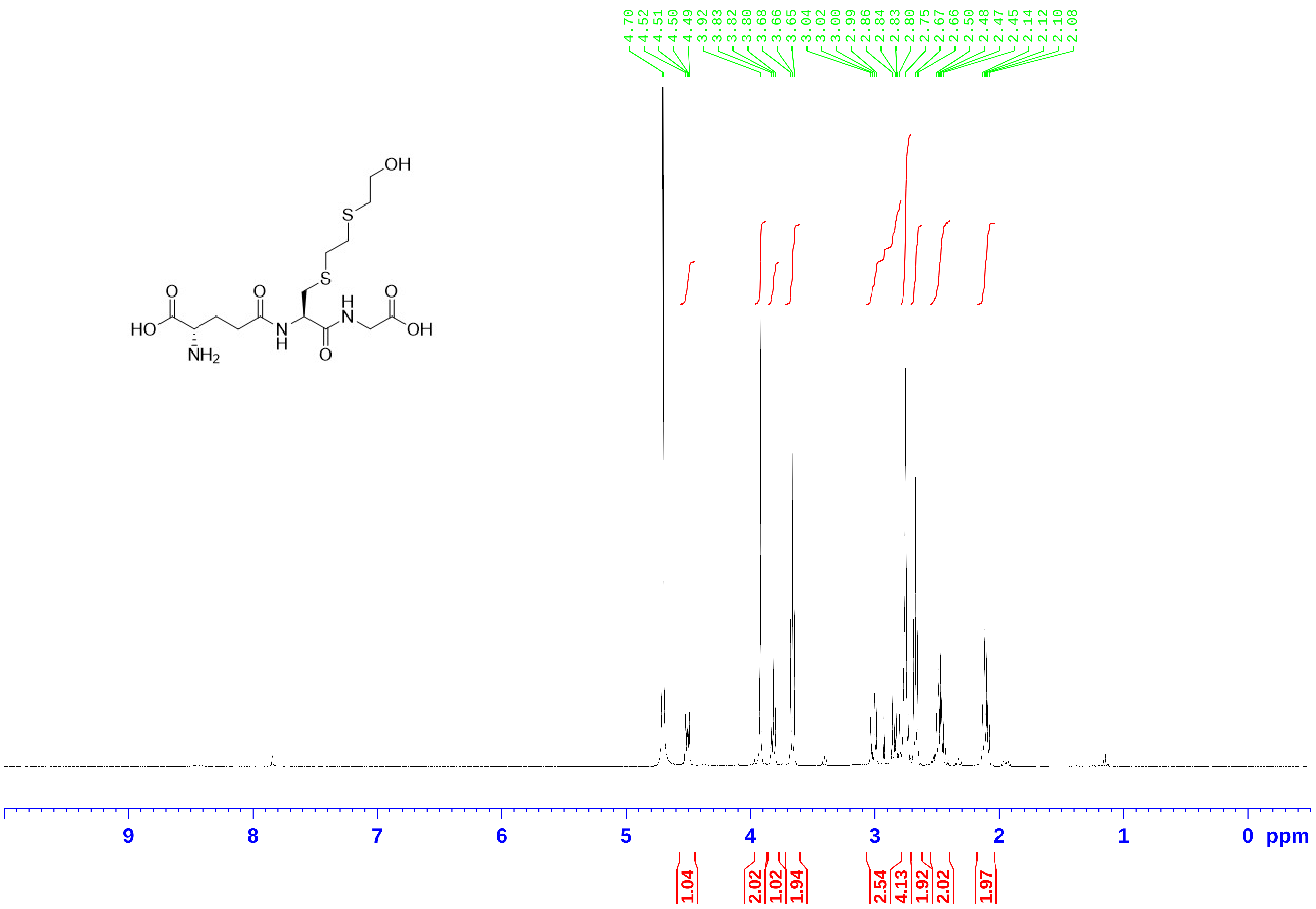

HO
OH
OH
NH2
S
S
O
N
H
4.70 4.52 4.51 4.50 4.49 3.92 3.83 3.82 3.80 3.68 3.66 3.65 3.04 3.02 3.00 2.99 2.86 2.84 2.83 2.80 2.75 2.67 2.66 2.50 2.48 2.47 2.45 2.14 2.12 2.10 2.08
1.04 2.02 1.02 1.94 2.54 4.13 1.92 2.02 1.97
9 8 7 6 5 4 3 2 1 0 ppm

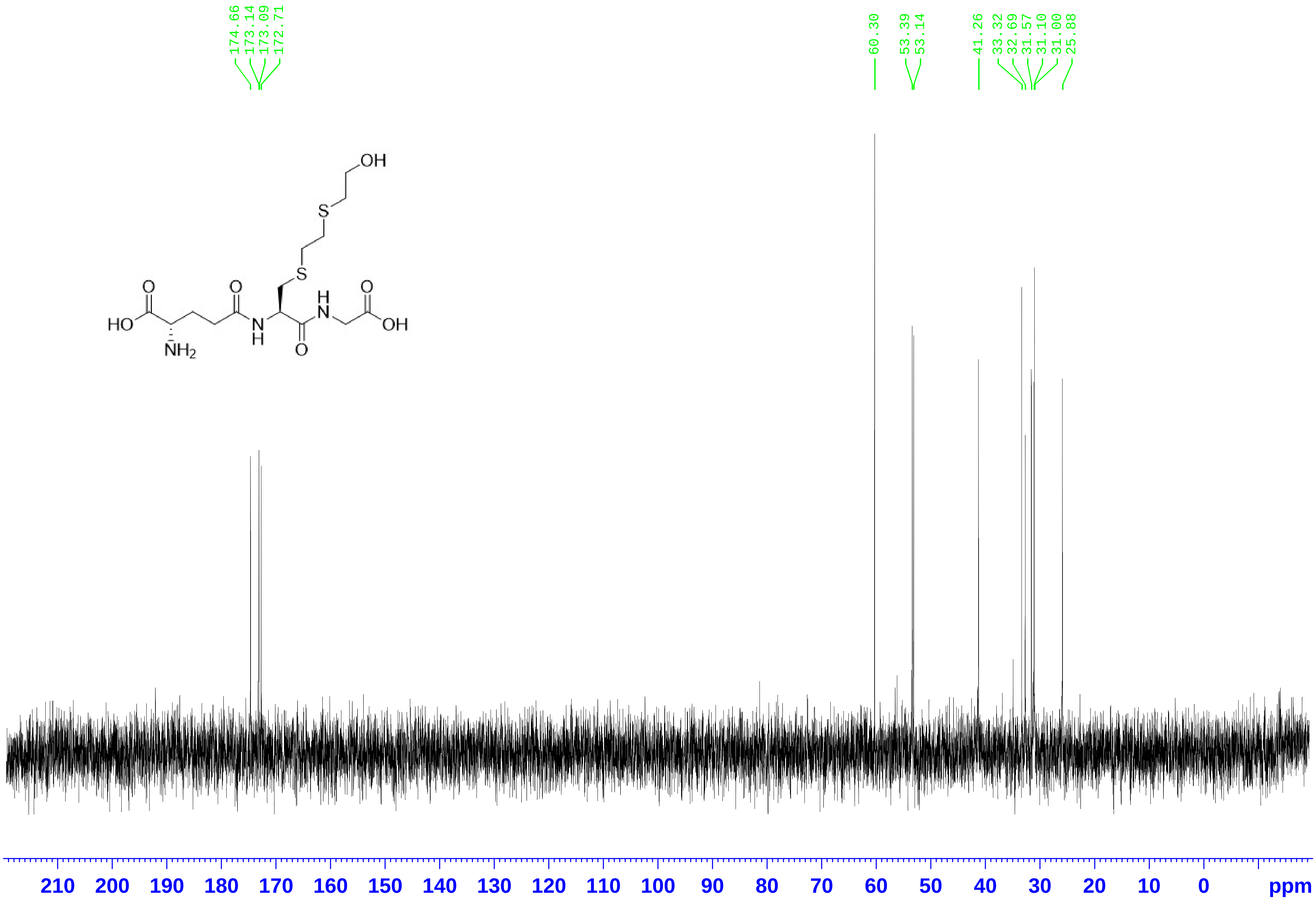
174.66
173.14
173.09
172.71
60.30
53.39
53.14
41.26
33.32
32.69
31.57
31.10
31.00
25.88
ppm

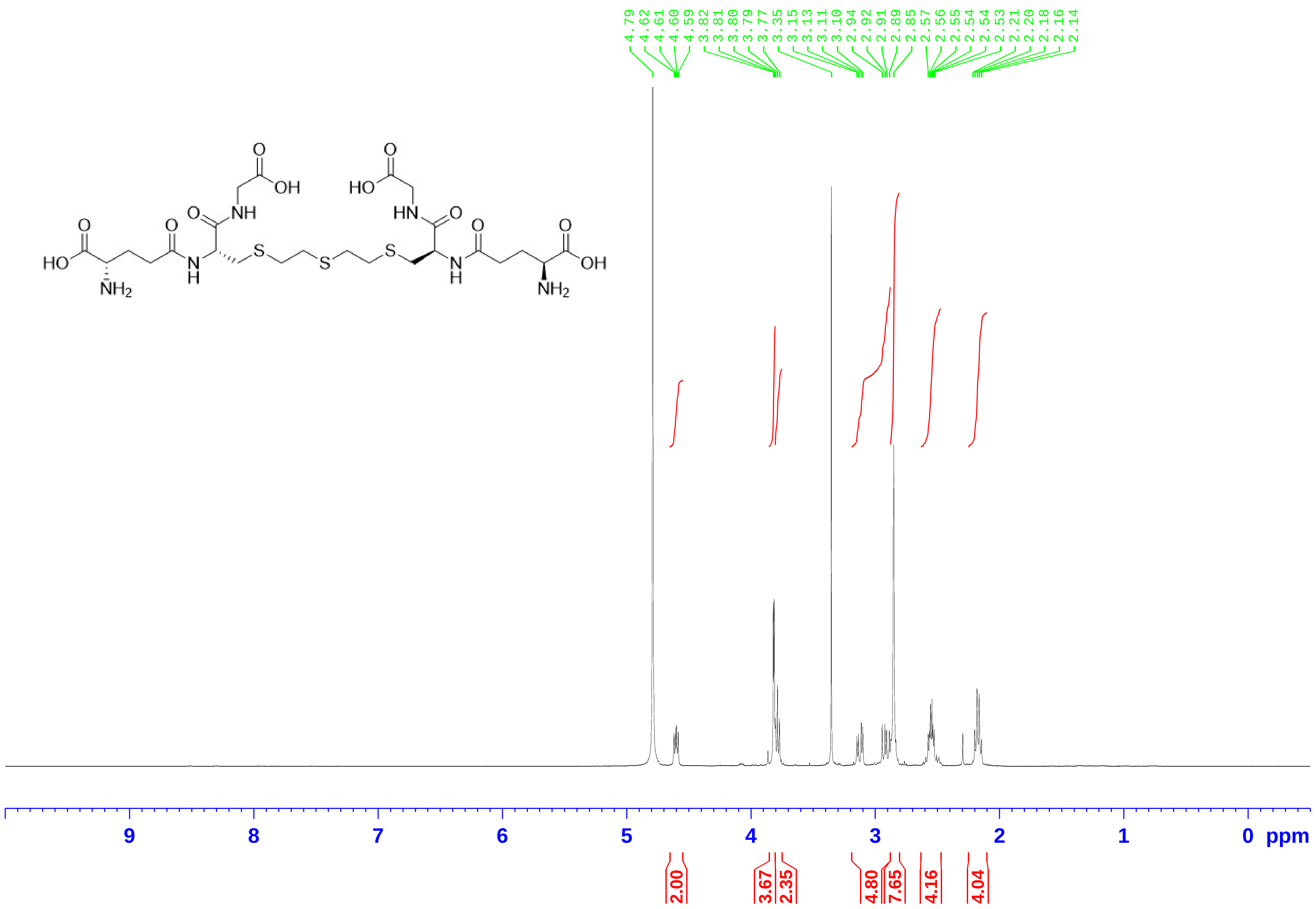
4.79
4.62
4.61
4.60
4.59
3.82
3.81
3.80
3.79
3.77
3.35
3.15
3.13
3.11
3.10
2.94
2.92
2.91
2.89
2.85
2.57
2.56
2.55
2.54
2.54
2.53
2.21
2.20
2.18
2.16
2.14
9
8
7
6
5
4
3
2
1
0
ppm
2.00
3.67
2.35
4.80
7.65
4.16
4.04

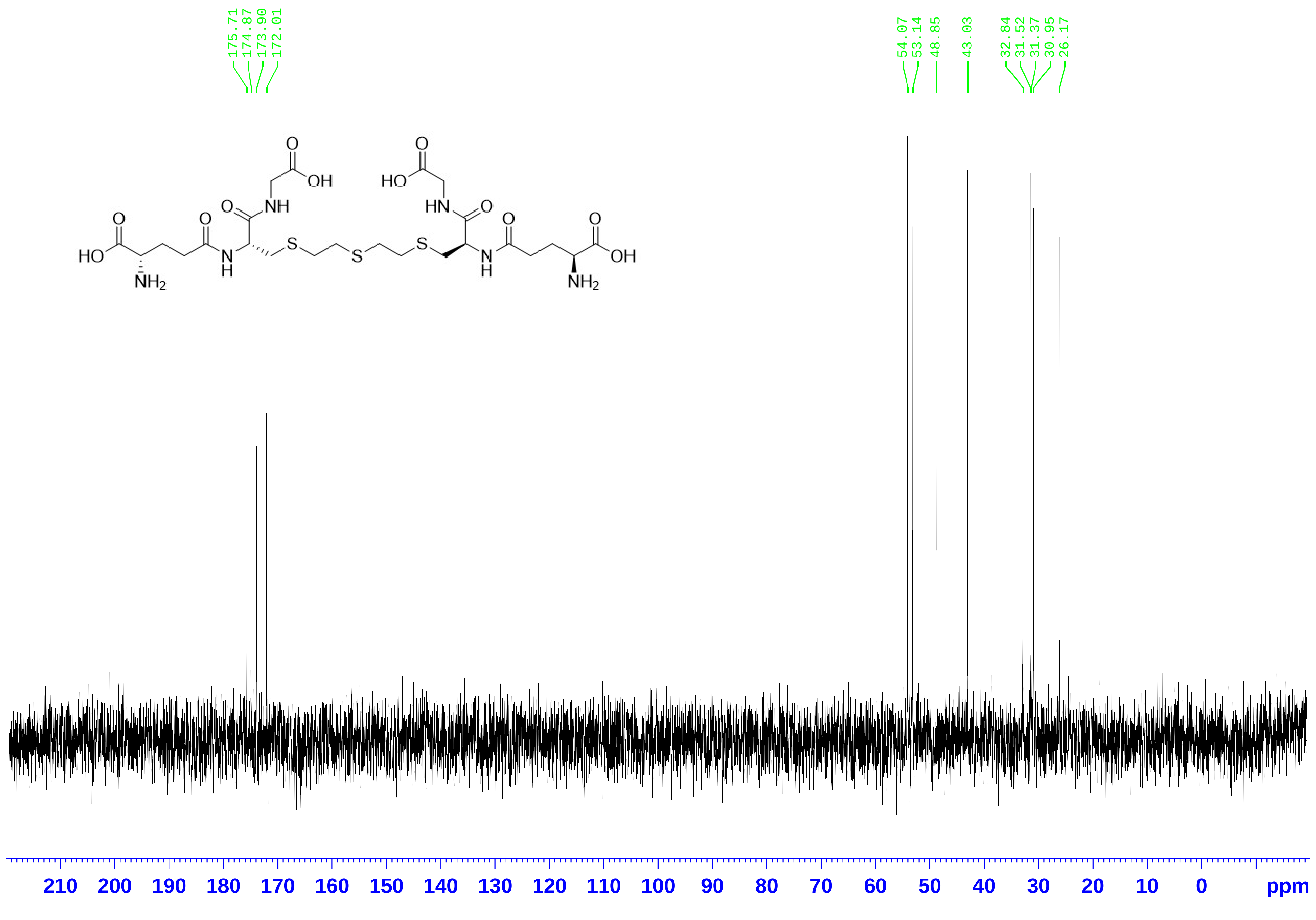
175.71
174.87
173.90
172.01
54.07
53.14
48.85
43.03
32.84
31.52
31.37
30.95
26.17
HO
NH2
O
N
H
NH
OH
S
HN
210
200
190
180
170
160
150
140
130
120
110
100
90
80
70
60
50
40
30
20
10
0
ppm

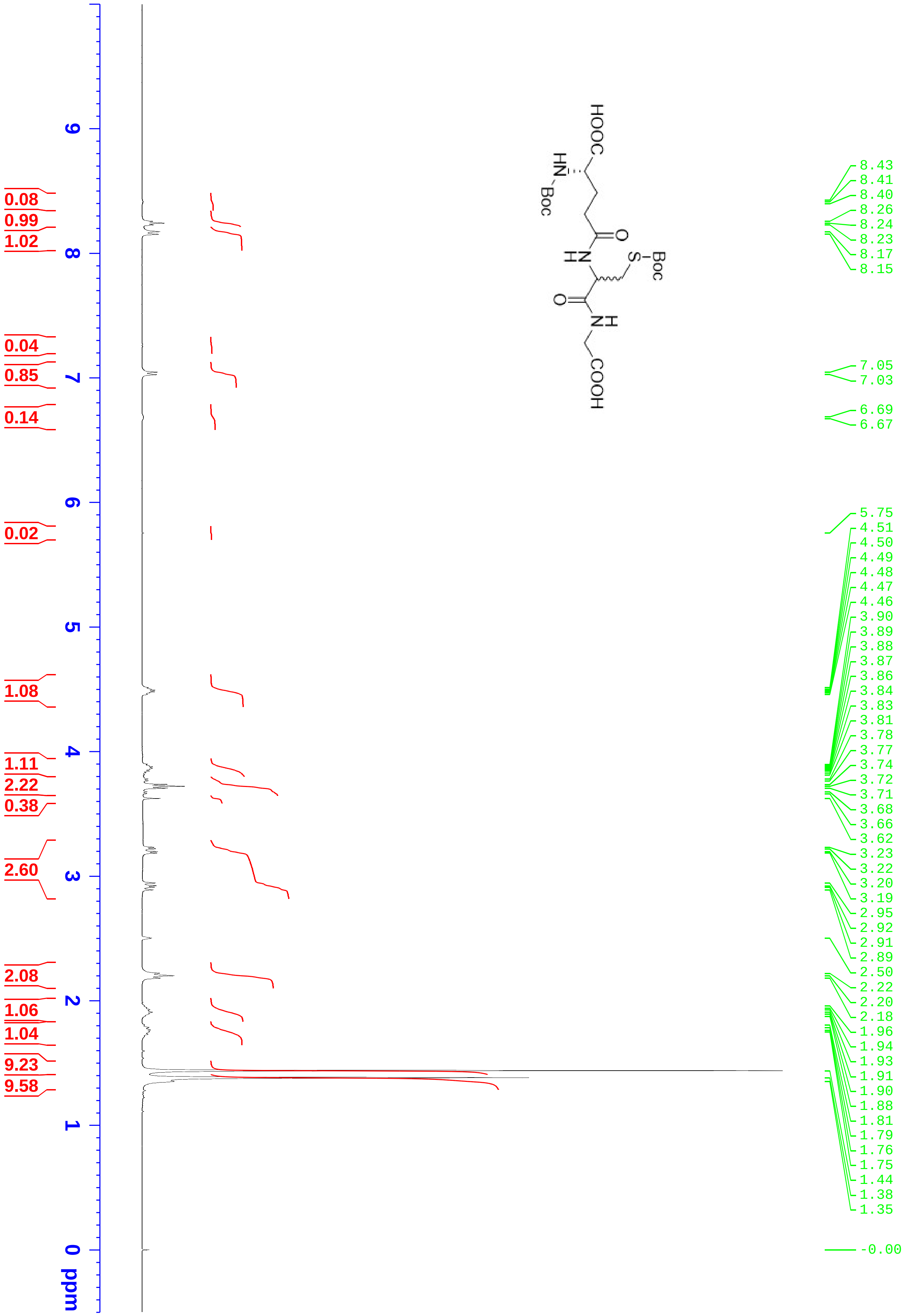

HOOC
HN
Boc
O
N
H
S
Boc
O
N
H
COOH
0.08
0.99
1.02
0.04
0.85
0.14
0.02
1.08
1.11
2.22
0.38
2.60
2.08
1.06
1.04
9.23
9.58
9
8
7
6
5
4
3
2
1
0
ppm
8.43
8.41
8.40
8.26
8.24
8.23
8.17
8.15
7.05
7.03
6.69
6.67
5.75
4.51
4.50
4.49
4.48
4.47
4.46
3.90
3.89
3.88
3.87
3.86
3.84
3.83
3.81
3.78
3.77
3.74
3.72
3.71
3.68
3.66
3.62
3.23
3.22
3.20
3.19
2.95
2.92
2.91
2.89
2.50
2.22
2.20
2.18
1.96
1.94
1.93
1.91
1.90
1.88
1.81
1.79
1.76
1.75
1.44
1.38
1.35
-0.00

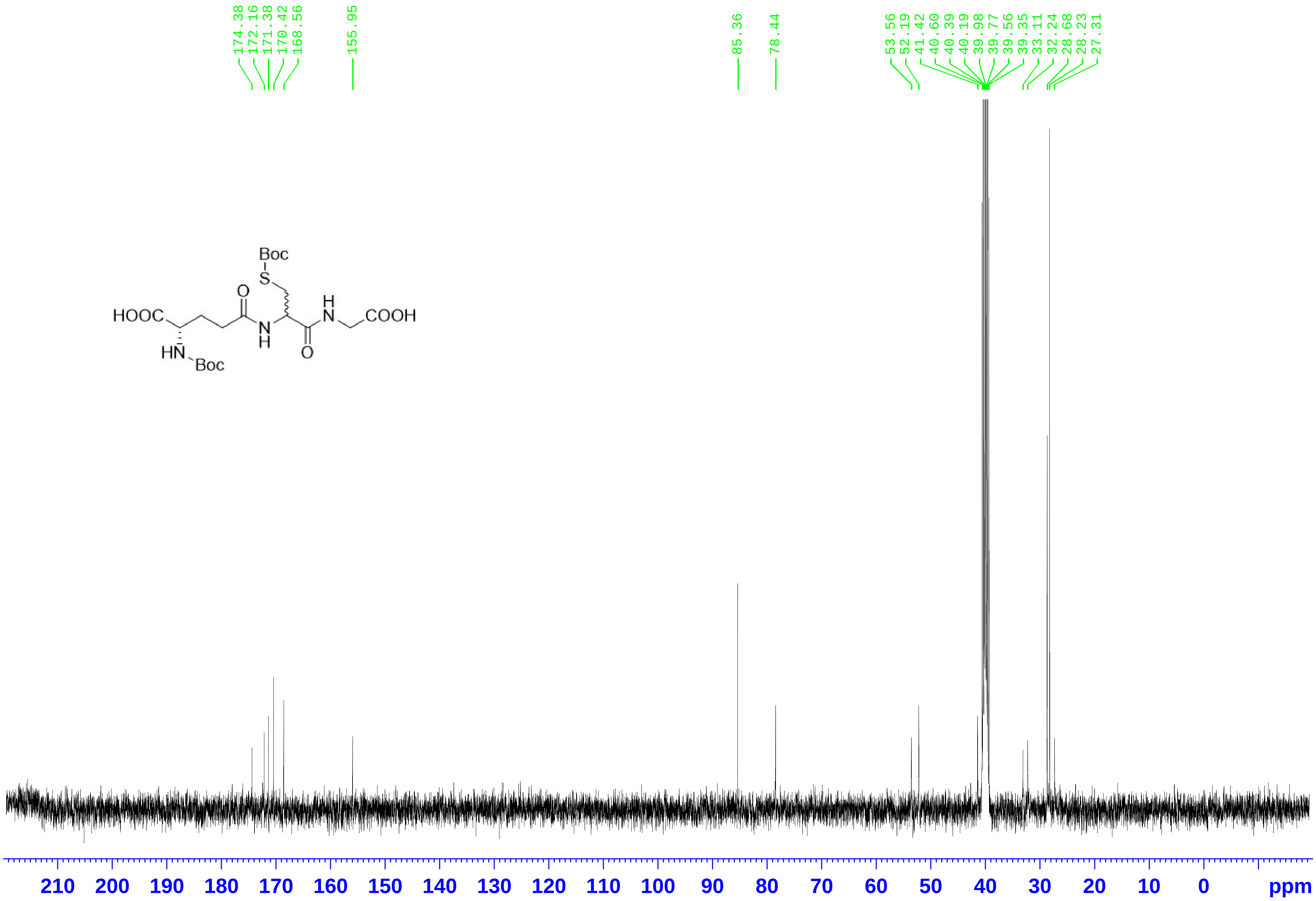
174.38
172.16
171.38
170.42
168.56
155.95
85.36
78.44
53.56
52.19
41.42
40.60
40.39
40.19
39.98
39.77
39.56
39.35
33.11
32.24
28.68
28.23
27.31
Boc
S
O
HOOC
N
H
H
N
COOH
O
HN
Boc
210
200
190
180
170
160
150
140
130
120
110
100
90
80
70
60
50
40
30
20
10
0
ppm

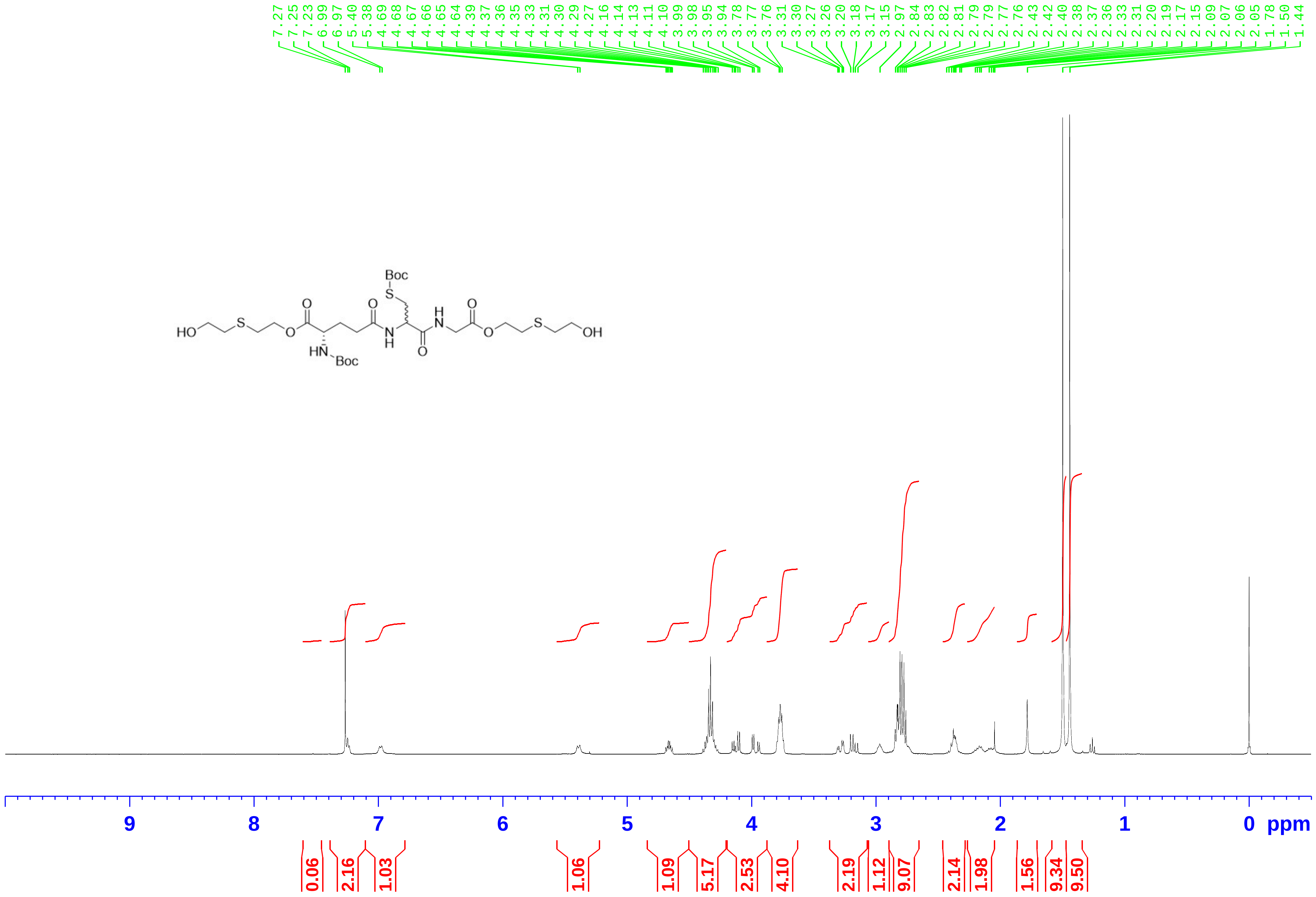

7.27
7.25
7.23
6.99
6.97
5.40
5.38
4.69
4.68
4.67
4.66
4.65
4.64
4.39
4.37
4.36
4.35
4.33
4.31
4.30
4.29
4.27
4.16
4.14
4.13
4.11
4.10
3.99
3.98
3.95
3.94
3.78
3.77
3.76
3.31
3.30
3.27
3.26
3.20
3.18
3.17
3.15
2.97
2.84
2.83
2.82
2.81
2.79
2.79
2.77
2.76
2.43
2.42
2.40
2.38
2.37
2.36
2.33
2.31
2.20
2.19
2.17
2.15
2.09
2.07
2.06
2.05
1.78
1.50
1.44
Boc
S
O
O
O
H
N
HO
S
O
N
H
O
O
S
OH
HN
Boc
9
8
7
6
5
4
3
2
1
0
ppm
0.06
2.16
1.03
1.06
1.09
5.17
2.53
4.10
2.19
1.12
9.07
2.14
1.98
1.56
9.34
9.50

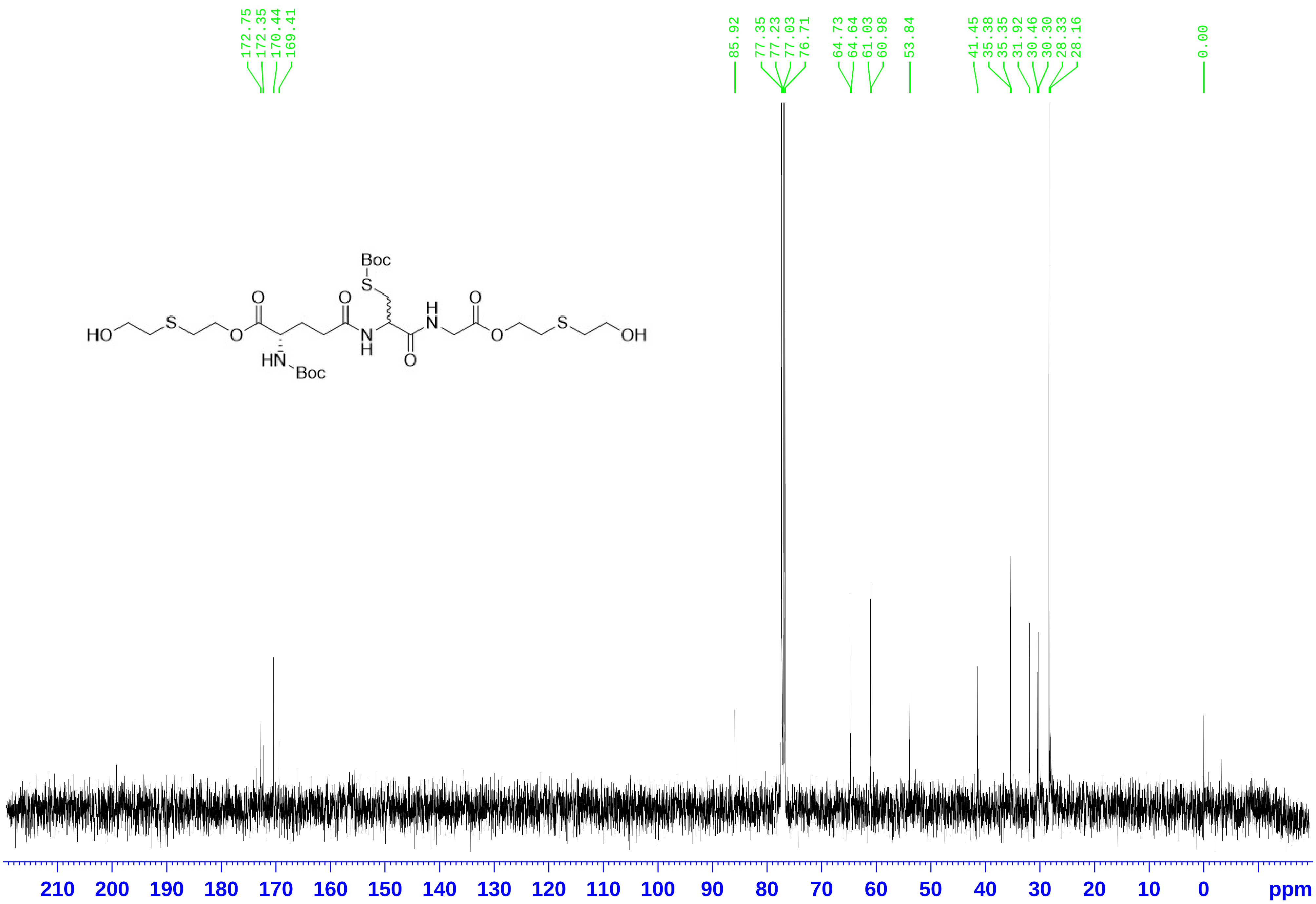
172.75
172.35
170.44
169.41
85.92
77.35
77.23
77.03
76.71
64.73
64.64
61.03
60.98
53.84
41.45
35.38
35.35
31.92
30.46
30.30
28.33
28.16
0.00
HO
S
O
HN
Boc
N
H
S
Boc
OH
210 200 190 180 170 160 150 140 130 120 110 100 90 80 70 60 50 40 30 20 10 0 ppm

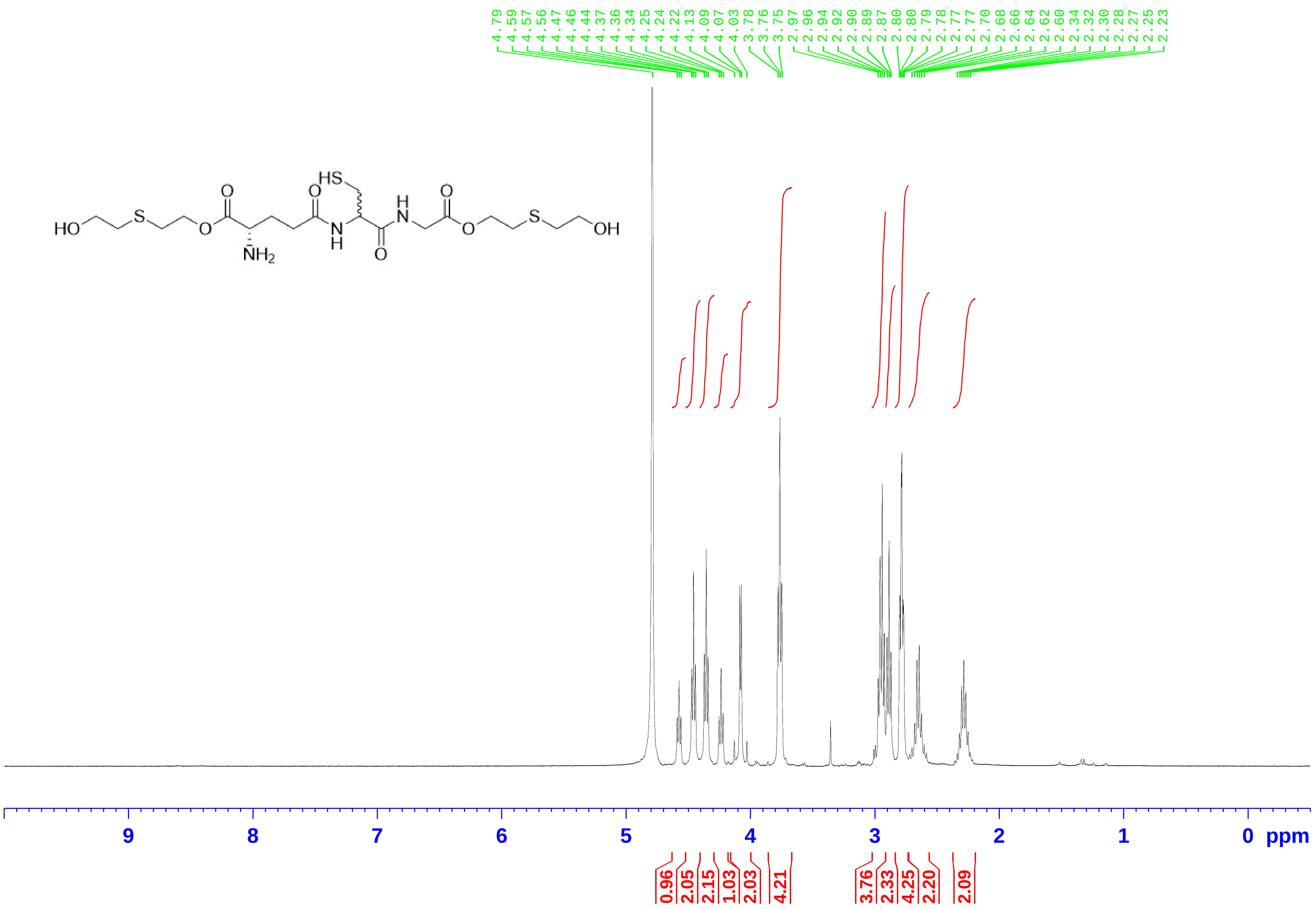
HO
S
O
O
NH2
O
N
H
HS
O
H
N
O
O
S
OH
4.79 4.59 4.57 4.56 4.47 4.46 4.44 4.37 4.36 4.34 4.25 4.24 4.22 4.13 4.09 4.07 4.03 3.78 3.76 3.75 2.97 2.96 2.94 2.92 2.90 2.89 2.87 2.80 2.80 2.79 2.78 2.77 2.77 2.70 2.68 2.66 2.64 2.62 2.60 2.34 2.32 2.30 2.28 2.27 2.25 2.23
9 8 7 6 5 4 3 2 1 0 ppm
0.96 2.05 2.15 1.03 2.03 4.21 3.76 2.33 4.25 2.20 2.09

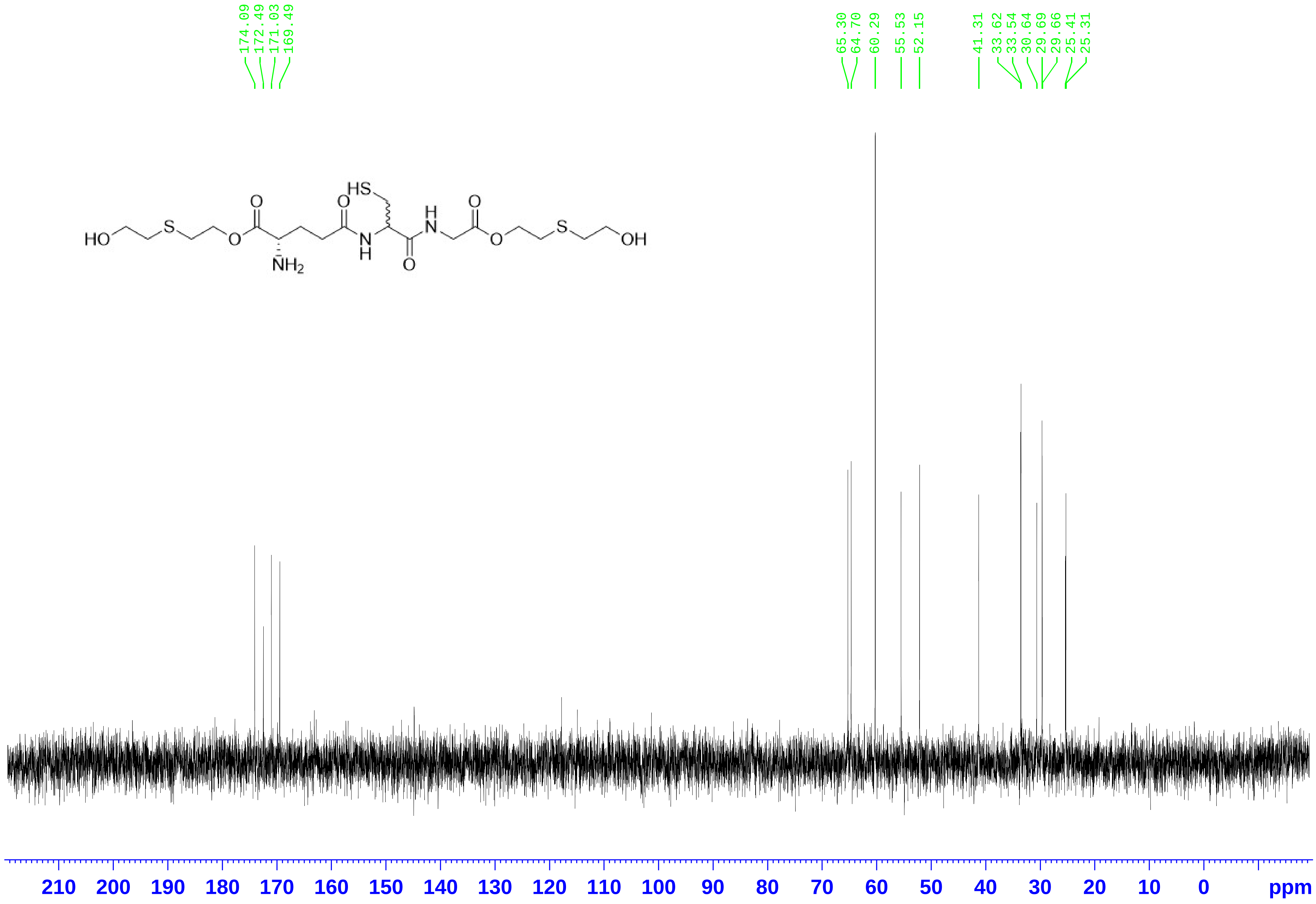
174.09
172.49
171.03
169.49
65.30
64.70
60.29
55.53
52.15
41.31
33.62
33.54
30.64
29.69
29.66
25.41
25.31
HO
S
O
O
NH2
O
HS
N
H
O
H
N
O
O
S
OH
210
200
190
180
170
160
150
140
130
120
110
100
90
80
70
60
50
40
30
20
10
0
ppm

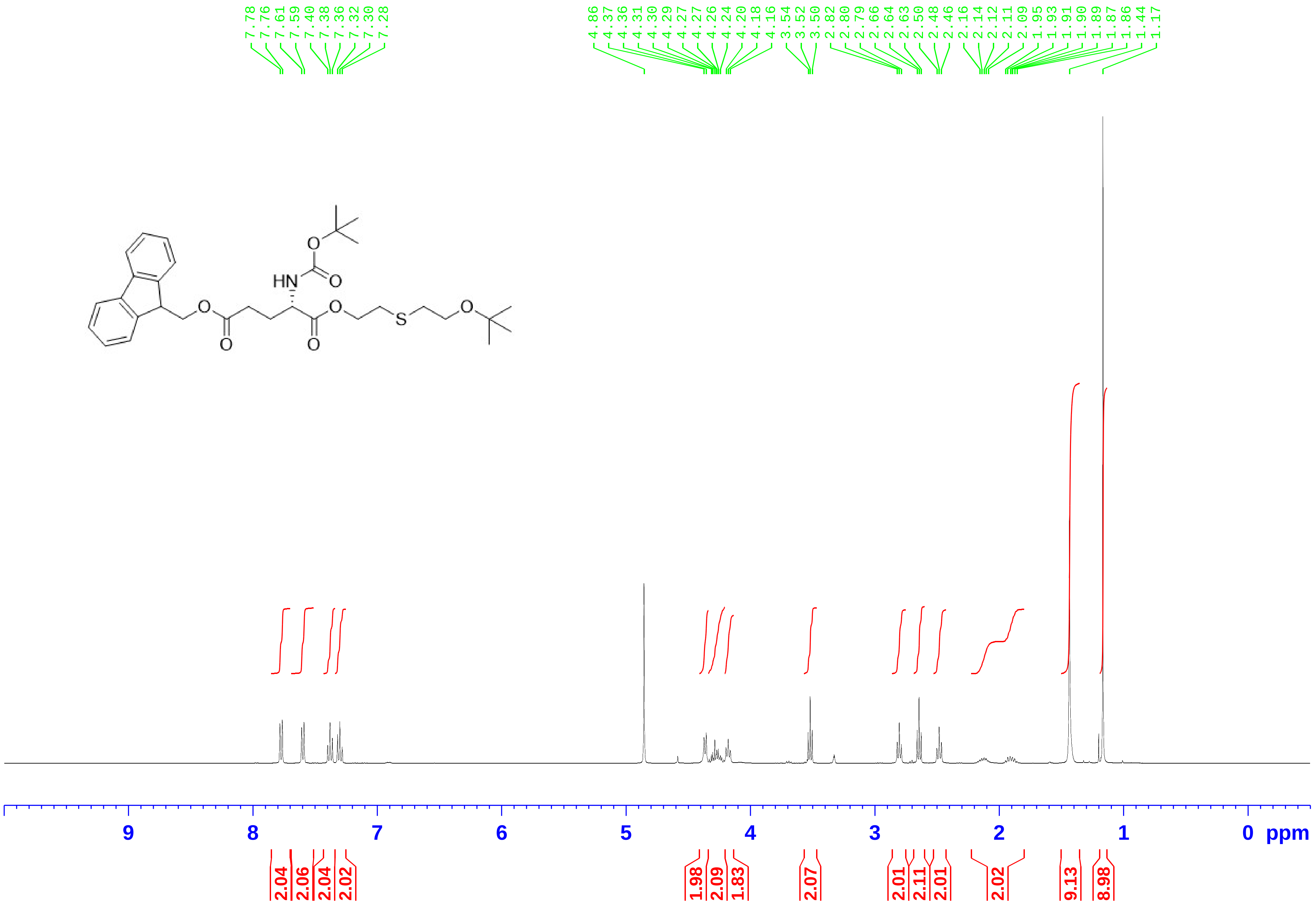

7.78 7.76 7.61 7.59 7.40 7.38 7.36 7.32 7.30 7.28
4.86 4.37 4.36 4.31 4.30 4.29 4.27 4.27 4.26 4.24 4.20 4.18 4.16 3.54 3.52 3.50 2.82 2.80 2.79 2.66 2.64 2.63 2.50 2.48 2.46 2.16 2.14 2.12 2.11 2.09 1.95 1.93 1.91 1.90 1.89 1.87 1.86 1.44 1.17
HN
O
S
9
8
7
6
5
4
3
2
1
0
ppm
2.04 2.06 2.04 2.02
1.98 2.09 1.83
2.07
2.01 2.11 2.01
2.02
9.13
8.98

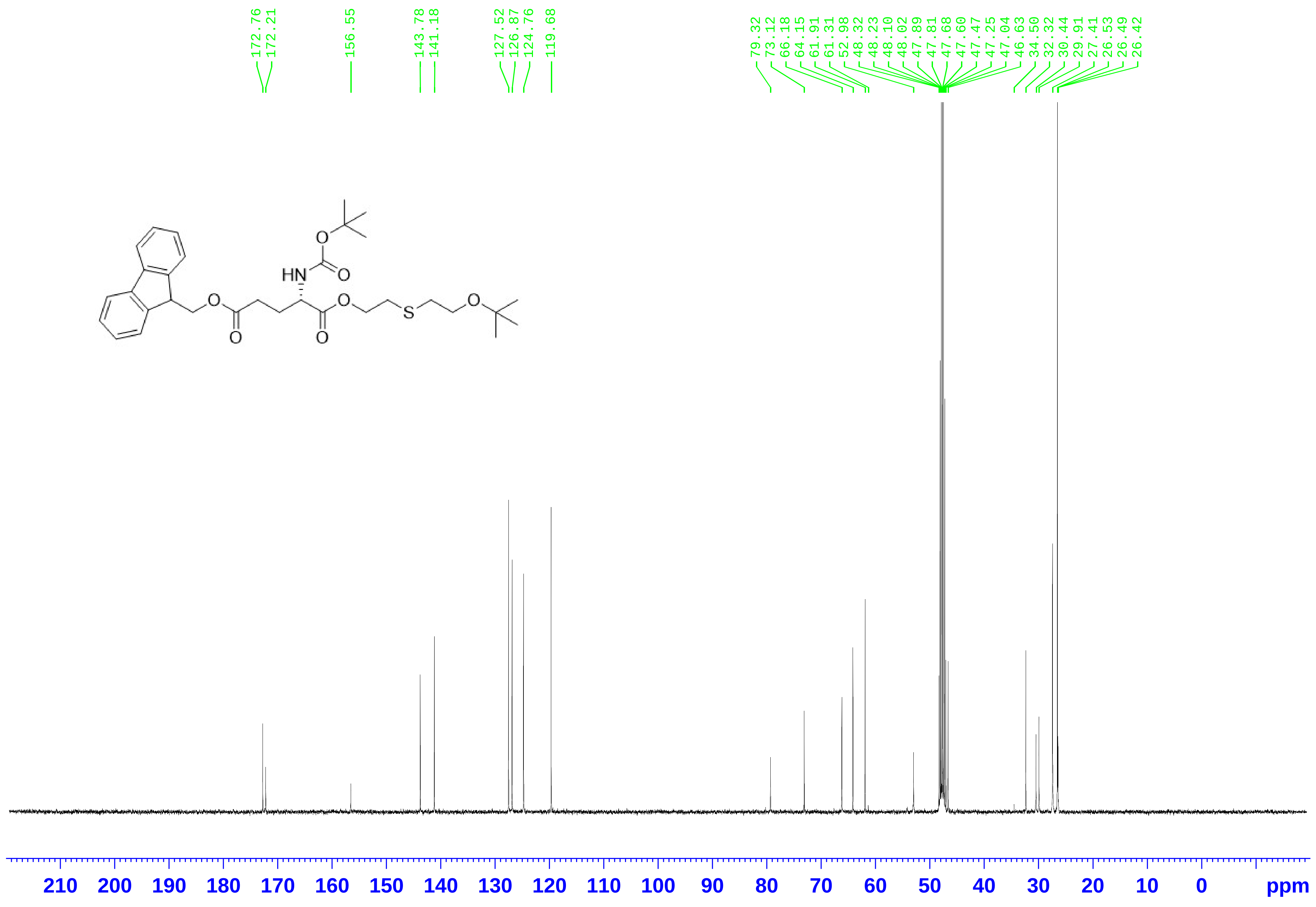

172.76
172.21
156.55
143.78
141.18
127.52
126.87
124.76
119.68
79.32
73.12
66.18
64.15
61.91
61.31
52.98
48.32
48.23
48.10
48.02
47.89
47.81
47.68
47.60
47.47
47.25
47.04
46.63
34.50
32.32
30.44
29.91
27.41
26.53
26.49
26.42
HN
O
S
210 200 190 180 170 160 150 140 130 120 110 100 90 80 70 60 50 40 30 20 10 0 ppm

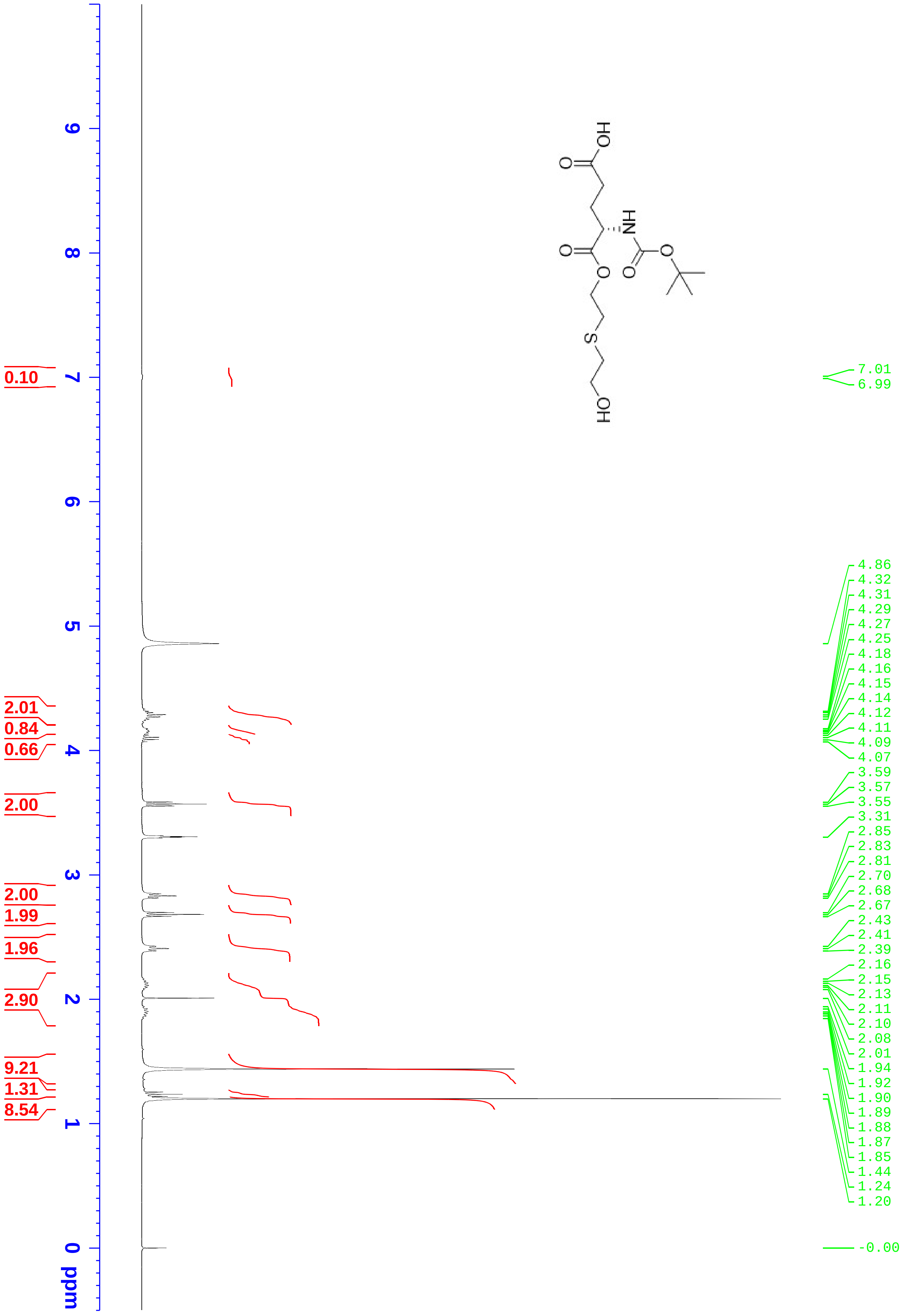

HO
O
O
NH
O
O
O
S
HO
7.01
6.99
4.86
4.32
4.31
4.29
4.27
4.25
4.18
4.16
4.15
4.14
4.12
4.11
4.09
4.07
3.59
3.57
3.55
3.31
2.85
2.83
2.81
2.70
2.68
2.67
2.43
2.41
2.39
2.16
2.15
2.13
2.11
2.10
2.08
2.01
1.94
1.92
1.90
1.89
1.88
1.87
1.85
1.44
1.24
1.20
-0.00
0.10
2.01
0.84
0.66
2.00
2.00
1.99
1.96
2.90
9.21
1.31
8.54
9
8
7
6
5
4
3
2
1
0
ppm

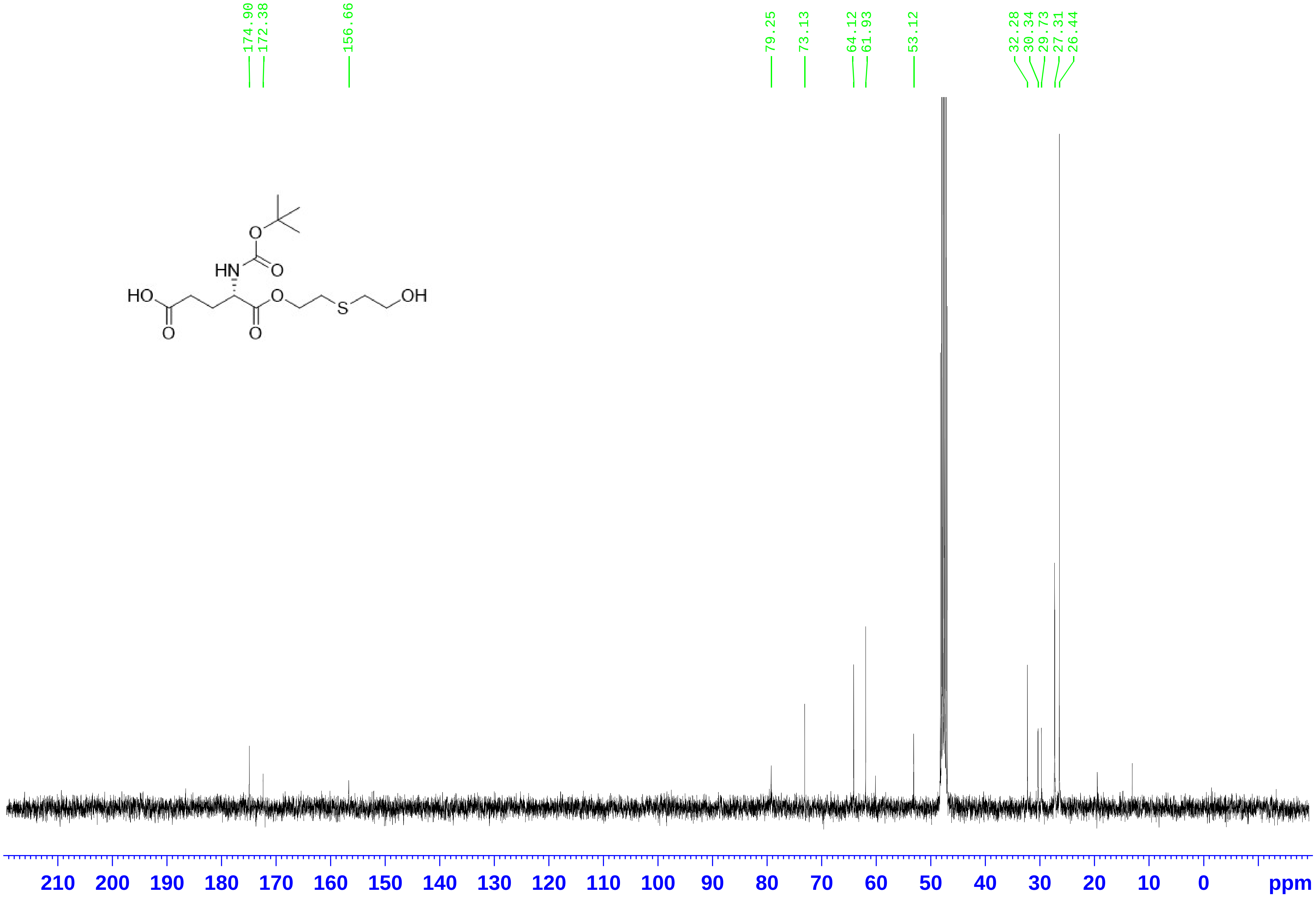
174.90
172.38
156.66
79.25
73.13
64.12
61.93
53.12
32.28
30.34
29.73
27.31
26.44
HO
O
HN
O
O
O
O
S
OH
210
200
190
180
170
160
150
140
130
120
110
100
90
80
70
60
50
40
30
20
10
0
ppm

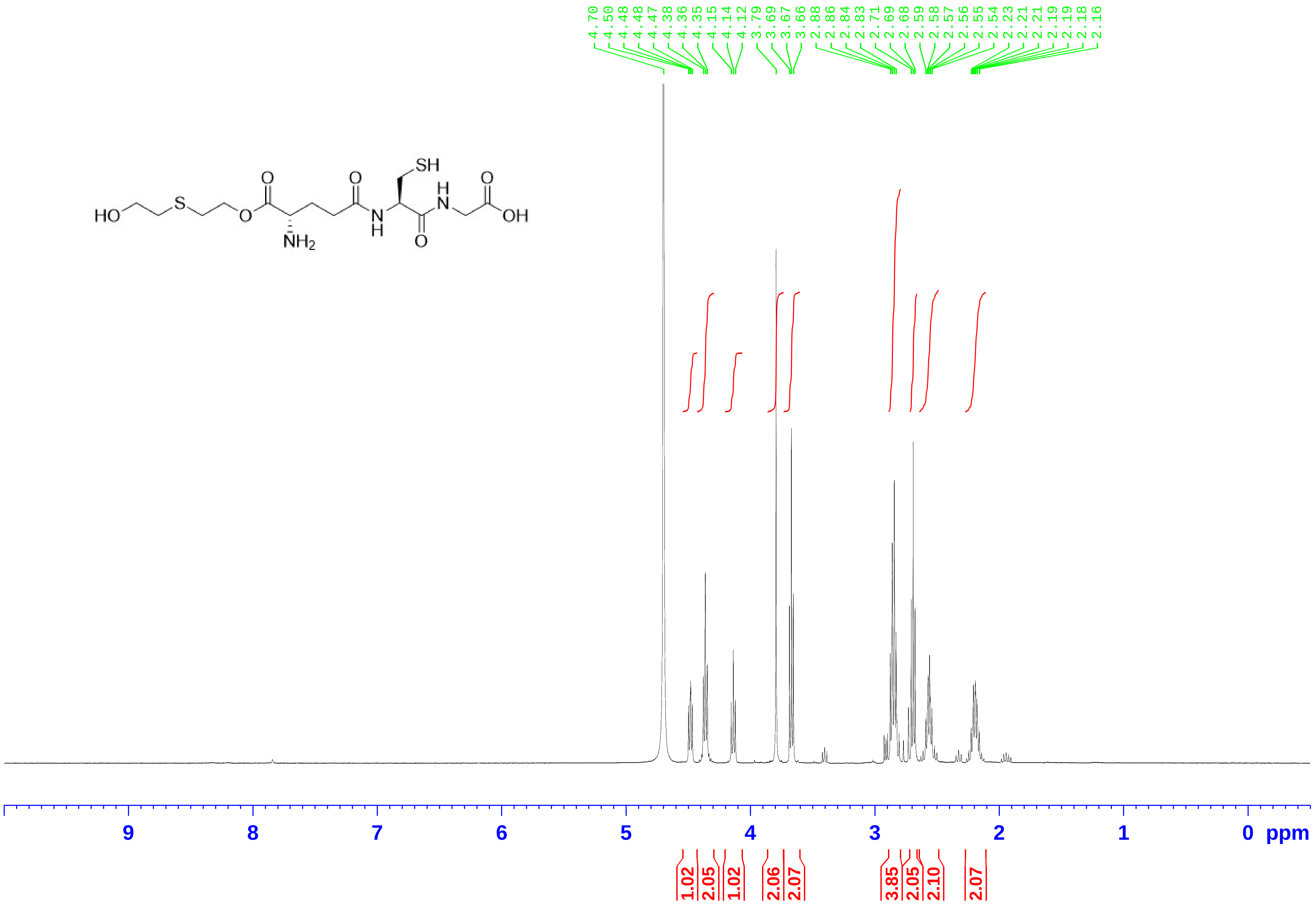
HO
S
O
O
NH2
O
N
H
SH
O
H
N
O
OH
4.70
4.50
4.48
4.48
4.47
4.38
4.36
4.35
4.15
4.14
4.12
3.79
3.69
3.67
3.66
2.88
2.86
2.84
2.83
2.71
2.69
2.68
2.59
2.58
2.57
2.56
2.55
2.54
2.23
2.21
2.21
2.19
2.19
2.18
2.16
9
8
7
6
5
4
3
2
1
0
ppm
1.02
2.05
1.02
2.06
2.07
3.85
2.05
2.10
2.07

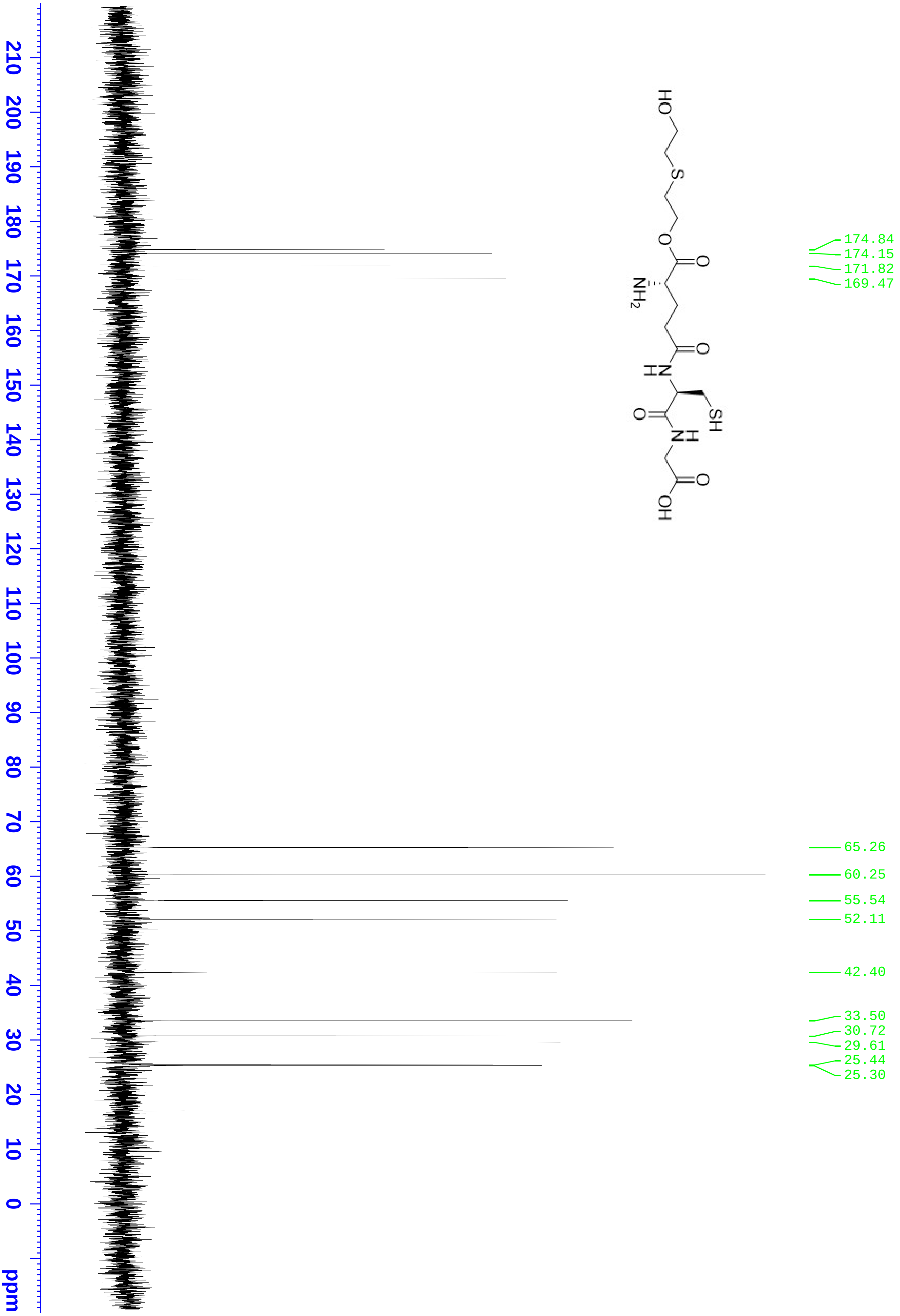
174.84
174.15
171.82
169.47
65.26
60.25
55.54
52.11
42.40
33.50
30.72
29.61
25.44
25.30
210
200
190
180
170
160
150
140
130
120
110
100
90
80
70
60
50
40
30
20
10
0
ppm
